# Support for temporary protection of displaced populations in the EU: A conjoint experiment

**DOI:** 10.1016/j.ejpoleco.2024.102601

**Published:** 2024-12

**Authors:** Michal Krawczyk, Andrea Blasco, Tomasz Gajderowicz, Marek Giergiczny

**Affiliations:** a Joint Research Centre of the European Commission, Brussels, Belgium; b Faculty of Economic Sciences, University of Warsaw, Poland

**Keywords:** Displaced populations, Conjoint experiment, Attitudes toward migrants, Ukraine, Asylum policies, Temporary protection

## Abstract

Millions of people were forced to flee Ukraine after Russia's invasion on February 24, 2022, one of the fastest displacements in decades. Citizens' response in EU countries (where most displaced Ukrainians arrived) has been considerably more positive than in past refugee crises. This study investigates several possible drivers of this difference. We conduct a large conjoint experiment in six EU Member States, eliciting willingness to provide temporary protection to hypothetical groups of future migrants whose characteristics we manipulate systematically. We find that all of the experimental variables make a difference. We observe a greater support for protecting groups consisting of relatively many children and many women rather than men. The region of origin and the religious affiliation play a major role. Finally, we see greater support for people fleeing a war rather than poverty or the adverse consequences of climate change. While all these effects are identified consistently across different groups of respondents (e.g., the respondent's religion played a limited role), effect sizes vary considerably between countries. Finally, we randomly manipulate which aspect of temporary protection (social housing, access to the labour market) is emphasised in our communication to the participants. We find this manipulation to have a limited effect on the public support for the policy.

## 1 Introduction

Millions of Ukrainians had to flee their country after Russia's invasion on February 24, 2022. This displacement, one of the fastest in decades, was predominantly directed towards EU Member States. Unlike in previous major refugee movements, the citizens of these countries responded very positively, with strong support for the incoming Ukrainian nationals (see De Coninck, 2022 for the comparison with Afghan nationals). On the political level, the overall support was reinforced with the decision to activate the Temporary Protection Directive (which was adopted following the Kosovo refugee crisis in 2001 but remained unutilised) and to adopt the Council Implementing Decision establishing temporary protection for displaced persons fleeing Ukraine.

Many commentators (Associated Press, 2022; Churruca 2022; Esposito, 2022) have linked these different attitudes to the tendency of public opinion to look more favourably towards certain groups of migrants and refugees than others, depending on their religion, ethnicity, and country of origin. The current displacement brings people from a traditionally Christian European country. By contrast, people escaping conflict in previous cases, such as in 2015–2016 and those crossing the Belarussian border in 2022, were mostly Muslims and predominantly from the Middle East.

These repeatedly confirmed effects of religion and ethnicity cannot be overlooked. In this project, however, we hypothesise that refugees are characterised on several other dimensions that also shape hosting populations’ attitudes towards them, as well as their support for relevant policy measures. These additional factors could help explain the discrepancy between past and current refugee crises. We confirm this hypothesis, quantifying the effect of each dimension. One critical issue is the (perceived and actual) high number of children among the displaced Ukrainian nationals. Another is a high share of women. Yet another is the reason for displacement; in particular, respondents might feel it is an obligation to support the victims of a war following an external (Russian) aggression widely covered in the media. By contrast, according to available statistics and the media portrayal, the Syrian refugees (the largest and most salient group in the 2015–2016 crisis, on which we thus focus our discussions) were much more commonly young (but adult) males. Moreover, the media showed “little connection between stories on migration and war reporting” (Joris et al., 2018).

The fact that different crises differ simultaneously on many dimensions means that the individual effects are nearly impossible to disentangle by secondary data analysis. To make the inference harder still, various interaction effects are plausible. For example, public opinion may be particularly disinclined to welcome Muslim men but not women (Ward, 2019). Likewise, if war is the reason for displacement, public opinion may expect men but not women to stay and defend their homeland.

To disentangle various factors that may shape the attitudes towards refugees, we conducted a choice-based conjoint experiment (a method referred to in other disciplines as a discrete choice experiment, Carson and Czajkowski, 2014). We presented study participants with different situations of hypothetical large groups of people forced to leave their country and seek protection in the EU. For each of these groups, we manipulated their key characteristics (gender composition, age composition, reason for displacement, etc.). We then asked participants to compare two situations and choose in which of them they would be more inclined to offer temporary protection, if any. We also manipulated the question the participants faced, highlighting different aspects of the type of protection afforded to the beneficiaries. This manipulation allowed us to investigate the effect of emphasising some key aspects of refugee assistance: providing access to public housing, access to the labour market, and access to medical care and education.

We recruited nearly 15,000 people from six countries for the experiment via an online survey administered by Valdari, Vicari & Associati (VVA) in Czechia, France, Germany, Hungary, Poland, and Romania. The survey aimed at a representative sample of the adult population in these countries using a sampling scheme based on demographic quotas for age, gender, education, geographic location, and employment status (See Appendix A).

Our analysis may contribute to a deeper understanding of the main factors influencing public opinion's willingness to accept and support refugees and its variability. In turn, our findings can be used to predict attitudes in possible future displacement crises, depending on the characteristics of the displaced and the reason for displacement. Our hope is that the study informs migration policies and communication surrounding migration, including preparedness programs for future refugee crises, especially in terms of preferable return policies, integration policies, and relocation policies. This is a very timely discussion, given the dynamic political landscape: the mass arrivals of Syrian nationals in 2015–2016 “threw the European asylum system into disarray” (Hatton, 2020) amidst bitter fights between Member States over the allocation of the displaced.

Refugees may bring many benefits to the hosting country (Pekkala Kerr and Kerr, 2011), particularly when they complement the locals in terms of distribution by age and skills (Quak 2019). These benefits are much less likely to come to fruition, however, when the refugees are greeted with mistrust and hostility. Aksoy et al. (2023) leveraged quasi-random allocation of refugees in Germany to find that negative attitudes toward migrants (as measured using geo-coded Twitter data) shape refugees integration success to the same degree as local unemployment rates do. As argued by Ruhs (2022), “public views constitute a soft feasibility constraint on effective and sustainable policies towards asylum seekers and refugees, and that a failure to take seriously and understand the attitudes of the host country's population can have a very damaging effect on refugee protection and migrants' rights in practice.” A better understanding of citizens' attitudes may thus improve acceptability of migration policy measures. It can also help anticipate and alleviate social tensions, i.a., by informing communication about migration and migration policies.

Our study builds upon extensive literature that we review in the subsequent section. Still, there are several features setting it apart from the most closely related work, i.e., conjoint experiments focused on attitudes towards refugees (or migrants in general) and related policy preferences. First, we have large, nationally representative samples from six EU Member States. The only study comparable in this respect is that by Bansak et al. (2016, 2023). One difference is that we place a special focus on Central and Eastern European countries that have seen a large number of displaced Ukrainian nationals in 2022 and are at the European Union's frontier; they are likely to remain particularly exposed to future inflows of displaced persons as the tensions in the Middle East and, especially, the former Soviet Union persist.

Second, Bansak et al. (2016, 2023) and many other studies have focused on asylum decisions for single individuals while we focus on attitudes towards a hypothetical, large group of individuals. This choice avoids some limitations of the single-applicant approach. First, asylum decisions (and individual-level recognition of circumstances warranting temporary protection) are made by state officials who are not elected, and so can be independent of public opinion's preferences. By contrast, activating temporary protection is always a political decision (Trauner and Valodskaite, 2022). Naturally, seeking re-election, politicians may be influenced by the currently prevailing public sentiment. This sentiment, in turn, is likely to be shaped by how the public perceives the characteristics of the group of refugees in question, including their socio-demographics. We thus believe our study is much more directly relevant for the policymaking process.

Second, asking respondents questions about groups of displaced individuals allows for identifying possible non-linear effects that would remain hidden in the single-applicant approach. For example, instead of distinguishing between a male and a female refugee, we elicit reactions to a fraction of women among the refugees (equal to 20% vs. 50% vs. 80%). It allows us to test if the effect of gender composition on popular attitudes towards refugee protection is constant or not. For example, it could be that upon reaching a certain threshold, further changes in the gender composition matter little (although in practice, as we will show later, we do not observe such non-linearity). The only other study we are aware of, which inquired about groups and so allowed for non-linearity was that by Ward (2019); as in typical single-migrant studies, however, the focus was on preference for a given group to be settled “in your neighborhood” rather than on preference for policies.

Unlike previous conjoint experiments on refugee policies, our study includes the monetary cost of the protection policy as an additional dimension in the experiment. This represents a unique feature, which adds to the study's realism, as accepting refugees requires allocation of resources. It also enables us to express participants' preferences in terms of willingness to pay which allows for a more straightforward interpretation of the trade-offs involved.

By contrast, we do not consider refugees' professions. The main reason is that it is naturally multidimensional: for example, Hainmueller and Hopkins (2015) considered as many as 11 different professions. Such diversity would be hard to reconcile with our design featuring a group, not a single refugee: while respondents can be asked to imagine one janitor, programmer or childcare provider, it is difficult to imagine a million refugees, all of them representing one specific occupation. Such an exercise would likely be awkward from the participant's viewpoint of and perhaps less useful from the viewpoint of study implications. Additionally, there is some evidence that, compared to other migrants, attitudes towards refugees are much less dependent on their labour-market characteristics (Lergetporer et al., 2018), although Bansak et al. (2016, 2023) found them to be important.

Another novelty is that we manipulate the task the participants faced, varying the emphasis on different rights that beneficiaries of temporary protection enjoy. Previous survey experiments suggest that people's attitudes towards refugees are influenced by how the crisis is portrayed (e.g. humanitarian vs. security problem, as in Getmansky et al., 2018 or using a benefit vs. victim vs. threat frame as in Liu, 2022). Here, we compare the baseline presentation of temporary protection (displayed to a control group) to the versions emphasising housing assistance and labour market access.

Finally, on top of eliciting choices, we also explicitly ask our respondents to judge the importance of various attributes (dimensions on which the hypothetical groups of future refugees differed). While this is sometimes done in choice experiments, these data are typically not even reported. Yet we believe they are highly interesting as they allow establishing willingness to admit to being influenced by various factors. In our case, for example, it could be that some respondents claim that the share of children among refugees affects their choices to a greater extent than it actually does. By contrast, they may, for example, want to hide their preference against Muslim migrants. Such comparisons between declared and revealed importance of different dimensions could thus yield a valuable insight into perceived social acceptability of different types of preferences concerning incoming refugees. They are also helpful from a methodological viewpoint. In particular, if declared and revealed importance were highly convergent, arguably there would be limited rationale to run complex and time-consuming choice experiments (instead of simply asking responders to rank different attributes).

We find that adult, male, Muslim (rather than Christian), African or Middle-Eastern (rather than Eastern European) migrants displaced by poverty or climate change (rather than war), and those whose protection is more expensive, are less likely to be supported than their counterparts, each dimension contributing at least a few percentage points. While religion and region of origin play a very large (but country-specific) role in choices, they are much less prominent in responders’ *declarations* as to how each dimension matters. Interestingly, our manipulation of the aspect of temporary protection which is emphasised, plays no significant role.

## 2 Related literature

While by now the number of surveys is too large to be thoroughly summarised here, the general attitudes towards the displaced Ukrainian nationals have been positive in most EU countries. If anything, pro-Ukrainian attitudes only strengthened in the course of invasion (Dražanová and Geddes, 2022). Accordingly, most respondents tended to declare that their governments could do more for Ukraine i.a. by providing additional benefits for the refugees or by taking diplomatic action such as support in peace talks (Staniszewski, 2022).

The positive attitudes are also reflected in helping behaviour; for example, thousands of people offered free accommodation (i.a. using services such as hospitalityforukraine.com). This contrasts with previous situations of this kind. For example, studies analysing the 2015 crisis show that in some cases, a sudden and large arrival of refugees may eventually result in hostility towards immigrants and minorities (e.g. Benček and Strasheim, 2016). Previous mass displacements also led to support for restrictive asylum and immigration policies (Hangartner et al., 2019), associated with the rise of populist right-wing parties (Steinmayr, 2021), typically leveraging Euroscepticism (Stockemer et al., 2020).

Existing literature suggests the religious differences between Ukrainians and Syrians as a plausible reason for this difference. Adida et al. (2019) conducted a conjoint experiment using a representative sample of 1800 US adults. In the wake of the 2015–2016 crisis, they provided study participants with pairs of hypothetical refugee profiles differing in terms of age, gender, profession, English language proficiency and, importantly, religion (Muslim vs. Christian). They asked them to rate, on a seven-point scale, the willingness to admit a given individual to the US (and, additionally, to choose from among each pair). Perhaps not surprisingly, high-skilled refugees fluent in English were most preferred, which may be understood in terms of their greater ability to contribute to the economy of the host country. However, religion was the single most important determinant, with negative attitudes towards Muslims prevailing in all demographic subgroups. This effect is likely due to perceived cultural threat. While association with (political) violence could have been a plausible alternative, Adida and colleagues found no interaction between gender and religion; if the association of Islam with terrorism was the driver, we would expect predominantly male Muslims to be less preferred (Ortbals and Poloni-Staudinger, 2018). However, Ji et al. (2021) did find an interaction: compared to their female compatriots, male Syrians but not male Liberian migrants trigger more negative attitudes among US-based Amazon Mechanical Turk workers (Ji et al., 2021). This is consistent with male but not female Muslims being perceived as a physical threat. Robust anti-Muslim sentiment was also reported in Europe (Bansak et al., 2016). In a large international sample they reached out to in the wake of the Syrian crisis, the authors found this pattern of preferences to be very stable across countries and participants’ individual characteristics.

Helbling and Traunmüller (2020) tried to dig deeper into anti-Muslim attitudes. On top of denomination, they also manipulated religious behaviour (non-practicing vs. devout vs. radical). It turned out to be of importance, with religious fundamentalists (Muslims, but to a weaker extent also Christians) triggering the strongest resentment among their British respondents.

Perhaps not surprisingly, the effect of religion may be reversed in a predominantly Muslim country. Alrababa'h et al. (2021) found their Jordanian participants to give lower ratings to Christian (and Alawite Muslim) profiles compared to the Sunni Muslim benchmark. This study is also unique in its focus on the case of a challenge incomparable to that experienced in Europe or the US: Jordan is a developing country with a very large number of refugees (exceeding three million, the total population of Jordan being just 10.5 million, World Bank, 2022).

Another key variable, preference for female refugees, was noted by many authors including Hainmueller and Hopkins (2015) in the US, by Bansak et al. (2016, 2023) across European nations and by Findor et al. (2022) in young Slovak adults. Gender preference has been linked to marriage prospects (Denney and Green, 2021): some citizens of the host country may perceive some refugees as potential romantic partners. Because a large majority of people are (exclusively or predominantly) heterosexual, this would mean an interaction effect, with men but not women showing more positive attitudes towards female refugees. This is typically not observed, however, perhaps due to an opposing effect: women may be more prone to perceiving male migrants as potential aggressors, making them prefer females as well.

Much less is known about the extent to which attitudes towards refugees or migrants in general may be affected by their age, specifically by some of them being minors. While there is some research on perception of refugee children per se (Plener et al., 2017; Aydoğdu and Gürsoy, 2021; Tercan et al., 2021), the only direct comparison of children and adult refugees in a conjoint experiment we are aware of is that of Alrababa'h et al. (2021) who found more positive attitudes towards Syrians refugees with children than those without children in their Jordan study.

Another dimension we manipulate is the reason for displacement, comparing economic migrants to climate change migrants and war refugees. This is motivated by the literature pointing out refugees are perceived differently from economic migrants (Abdelaaty and Steele 2022), a distinction which may have deepened in the European public debate during the 2015 crisis (Czymara and Schmidt-Catran 2017 found such an effect in Germany). One reason behind the diverging attitude is that economic migration tends to be perceived as voluntary, whereas displacement due to war or persecution is perceived as forced, triggering stronger support (Verkuyten et al., 2018). Climate change is also beyond the control of an individual. However, since migration may not be naturally perceived as the only way of avoiding its disastrous consequences, climate change migrants would be expected to occupy an intermediate position, being more acceptable than economic migrants but less acceptable than war refugees. This hypothesis formulated by Arias and Blair (2022) was supported by the data they collected in Germany and the US. In a conjoint experiment, the respondents were shown nine pairs of migrant profiles, in each case being asked to choose one of them to be admitted for settlement in their state. Compared to economic reasons of migrating, environmental reasons increased the probability to be preferred by 3.5–4 percentage points in the US and by 6–8 percentage points in Germany. For persecution, the effect was twice as large: 7.6 in the US and 16.2 in Germany. In a follow-up study, Arias and Blair (2022) found that support for policies aiding climate migrants is closely linked to self-reported empathy, a confirmation of intuitive claims made i.a. by Bansak et al. (2016), Alrababa'h et al. (2021) and, importantly, Fraser and Murakami (2021) who showed that the preference for refugees over labour migrants is driven by humanitarian considerations. Similar observations of climate migrants being perceived as an intermediate case between economic migrants and war refugees were made in Denmark (Hedegaard, 2022).

Finally, our study is closely related to the, admittedly scarce, research on preference for migration policies. Four conjoint experiments are worth mentioning in this regard. Letki et al. (2022) manipulated five attributes of a possible policy. Their choice was inspired by previous research and the European Commission's New Pact on Migration and Asylum policy proposal. These five attributes were i) allocation of refugees among Member States, ii) EU border control, iii) right to work, iv) freedom of movement for asylum applicants, and v) the policy's yearly cost per capita. Letki et al. (2022) collected their data in Germany, Hungary and Poland between January 11th and February 11th, 2022 and then, with the same and some additional participants, between April 25th and May 12th. The remarkable observation was that the war had not affected stated opinions on migration and asylum policies.

In their study on Colombians’ preference on policies concerning displaced Venezuelans, Allen et al. (2022)were also interested in the effect of exposure to a large number of refugees. However, they could identify it by comparing across respondents, not over time (pre-vs. during the crisis, as Letki et al. did). They found that those who had less contact with Venezuelans (as well as those focusing on the economic aspect of the crisis only) tended to support more restrictive immigration policies.

Vrânceanu et al. (2022) systematically modified the key characteristics of the EU-Turkey deal, namely 1) Return of migrants from Greece to Turkey 2) EU financial support to help refugees in Turkey 3) Resettlement from Turkey to EU, 4) EU support to Greece to deal with migration and 5) Turkish controls of border with Greece. In a conjoint experiment conducted in Germany, Greece, and Turkey, they observed choices apparently reflecting a mixture of motivations. The fact that Greek and German respondents generally opted for stronger border controls while those in Turkey for greater support from the EU can be explained in terms of national self-interest. However, humanitarian considerations were the most plausible motive preference for pro-refugee policy features such as rejecting “pushbacks”.

Finally, Jeannet et al. (2021) asked the respondents to choose between refugee policies differing in terms of limit of asylum applications, resettlements to respondent's country, possibility that the refugee (will) be sent back to an unsafe country, family reunification arrangements, centralisation of decision making, and financial solidarity. Intriguingly, they found similar preferences (balancing refugees' rights with constraints on hosting countries' expenses) across all countries of their diverse sample (Austria, France, Germany, Hungary, Italy, Poland, Spain, and Sweden).

## 3 Design

As for data collection strategy, the Discrete Choice Experiment (DCE) was employed. This method is invaluable for deciphering complex preferences. DCEs are deeply rooted in the foundational utility-based theories of Lancaster and McFadden. Essentially, DCE simulates a marketplace where respondents make choices that provide insights into their underlying preferences. The method involves presenting individuals with sets of hypothetical scenarios, each with various attributes, and asking them to choose their preferred option. This approach has been widely recognized for its ability to detail the multifaceted nature of decision-making, acknowledging that consumers weigh both visible and hidden factors when making choices. This is the only stated preference method that is based on Random Utility Model; it has been argued that it is methodologically superior to other conjoint approaches (Louviere et al., 2010).

Our study involved a randomised online survey addressed at the general adult population. The questionnaire consisted of the following modules:

M1: “warm-up” demographics and choice experiment on the determinants of support for temporary protection.

M2: a perspective taking/perspective getting experiment to increase helping behaviour.

M3: questions concerning perceptions and past behaviours towards the displaced Ukrainian nationals.

M4: attitudes towards immigration, political opinions, religious beliefs.

M5: remaining demographics.

In this paper, we focus on M1, with selected questions from M3-M5 serving as control variables. Separate publications will focus on M2 and M3 respectively. Because of the fixed order, these modules could not have any effect on the responses provided in M1.

In M1, the respondents were asked to consider a group of persons seeking temporary protection in the EU. The size of the group was specified as 1 displaced person per 500 residents of the country (which would correspond to nearly 1 million refugees EU-wide). Temporary protection meant that the displaced persons would receive.

1. … a residence permit, medical care, and housing in [Country] for a year. [Housing Treatment]
2. … a residence permit, medical care, and access to the labour market in [Country] for a year. [Labour Treatment]
3. … a residence permit and medical care in [Country] for a year. [Control Treatment]

These three definitions were randomised between the subjects. Each group of persons was characterised by six attributes, see Table 1 for a definition of each dimension and Table 2 for their possible levels. Table 1 was also shown to participants.

**Table 1:** Description of attributes.

Country	Country of origin
Displaced by	The reason for which they had to flee their country of origin
Fraction of women	The fraction (percentage) of women among the displaced adults
Fraction of children	The fraction (percentage) of children below the age of 14
Religious background	Religious background
Yearly cost for you	This is the yearly cost, to you, of the policy of temporary protection of this group of displaced persons. Every policy has its costs that are generally covered with taxes that you and other citizens pay (fiscal costs).

**Table 2:** Attribute levels.

Attribute	possible levels
Country of origin	An Eastern European country such as Ukraine or Moldova [Eastern Europe]
	A Middle Eastern country such as Syria or Lebanon [Middle East]
	Sub-Saharan African country such as Nigeria or Ethiopia [Africa]
Displaced by	poverty in their country [Poverty]
	climate change making their country uninhabitable [Climate]
	a war in their country [War]
Fraction of women	20 out of 100 adults are women [Women_20]
	50 out of 100 adults are women [Women_50]
	80 out of 100 adults are women [Women_80]
Fraction of children	10 out of 100 are children [Children_10]
	30 out of 100 are children [Children_30]
	50 out of 100 are children [Children_50]
Religion	90 out of 100 are Christian [Muslim_10]
	45 out of 100 are Christian, 45 are Muslim [Muslim_45]
	90 out of 100 are Muslim [Muslim_90]
Yearly cost for you	5, 10, 20, 40, 70, 100 euro (DE; for other countries, these amounts were converted into amounts in local currency with the same purchasing power and rounded)

The levels were chosen to correspond to relevant distributions of recent large waves of displaced people arriving in Europe. The Country of origin was not specified; instead we pointed at a group of countries, such as those in the Middle East, broadly sharing some characteristics (geographic location, language, culture, religion), accompanied by prominent examples (e.g. Syria or Lebanon) of countries that, historically, have sent migrants to the European Union, to help our readers make a connection. We recognise that such broad categories may represent an imperfect proxy for some specific countries belonging to them; this design choice was a compromise between the limitation of the number of levels of an attribute that could be accommodated and the attempt to make the estimates relevant for a future crisis that might lead to a large number of refugees from almost any country.

As for the costs, the estimates diverge considerably and the amounts certainly differ across EU countries. However, the range we used corresponds with our participants’ own estimates (see Q19 in Appendix B). In particular, the median estimate of daily costs of protecting one Ukrainian person was about 50 or about 80 euro depending on the country; this corresponds to 36–60 euro per year per inhabitant, thus an amount near the middle of our range.

In each case, the respondents were asked to consider two groups and indicate whether they would rather support temporary protection for Group A, Group B, or none, as displayed in Table 3. Six such tables were displayed to each participant, following a Bayesian efficient design generated using Ngene software (ChoiceMetrics, 2014), with the restrictions that (a large group of) refugees from an Eastern European country cannot be 90% Muslim and the refugees from a Middle-Eastern country cannot be 90% Christian. We are aware that Lebanon (as well as Egypt) has a notable Christian population and Syria had one before the war. Still, the belief that “all Arabs are Muslim and all Muslims are Arabs”, essentially identifying the “Muslim/Islamic world” with the Middle East, is often mentioned as a “common misconception” both in academic literature (e.g. Sammut et al., 2018) and popular sources (e.g. islamreligion.com). Although hard evidence on the prevalence of this myth in Europe seems hard to come by, we preferred to avoid the Middle East/90% Christian and Eastern Europe/90% Muslim combinations, fearing that many participants would find such a scenario less realistic.

**Table 3:** A typical screen in the choice experiment.

	Situation A	Situation B	None
Country of origin	An Eastern-European country such as Ukraine or Moldova	A Middle Eastern country such as Syria or Lebanon	I wouldn't support Temporary Protection in any of these two situations
Displaced by	A war in their country	Poverty in their country	
Fraction of women	50 out of 100 women	20 out of 100 women	
Fraction of children	10 out of 100 children	10 out of 100 children	
Religious background	90 out of 100 Christian	90 out of 100 Muslim	
Yearly cost for you	20 EUR	10 EUR	
Your decision:	O	O	O

The order of the rows was randomised between-subject to control for attribute order effects (but, to facilitate the task, each respondent saw the same order in the six tables she or he faced). Moreover, each participant was assigned to one of six groups facing different choice tasks. All between-subject randomizations were mutually independent and stratified by country.

After the Discrete Choice Experiment, participants were asked to rate each dimension on a scale from 0 to 10, where 0 meant that the dimension was not important to them at all when they were making their decisions, while 10 meant it was extremely important. If participants were fully conscious of their preferences and open about them, we would expect these declarations to correspond to importance of each dimension as estimated from the choices in the main task. If that was the case, in the future, the (more complex) procedure of choice experiments could possibly be replaced by direct questions in similar research. However, in practice, it may turn out that relative importance of dimensions differs depending on whether it is revealed implicitly vs. when it is openly declared.

The entire questionnaire can be found in Appendix B, while Appendix C covers the econometric strategy.

## 4 Hypotheses

Based on existing literature, we pre-registered several hypotheses (see https://www.socialscienceregistry.org/trials/9682) regarding determinants of participants' choices. Concerning the hypothetical refugee characteristics, we expected that populations that could be perceived as more deserving, less culturally and physically distant, and less threatening, will be more likely to be offered protection. Specifically, we hypothesised that willingness to pay (WTP) for policies protecting groups displaced by war will be high compared to the case of poverty (with climate change occupying an intermediate position), as proposed by Arias and Blair (2022) and others. We further expected that, in line with findings of Bansak et al. (2016, 2023) among others, WTP to support groups of refugees coming from a European country, with a high fraction of Christians, women, and minors, will be higher than in the case of complementary levels of these variables. Concerning the between-subject treatments, we hypothesised a heterogeneous effect of emphasising that the refugees would have access to the labour market; specifically, we expected relatively lower support for protection among the less-educated participants who would be more likely to feel threatened economically (O'Rourke and Sinnott 2006).

## 5 Sample and procedures

The experimental survey was programmed and distributed using the contractor's proprietary software, which ensures similar user experience regardless of the respondent's device and allows handling multiple language versions.

The master English-language version was developed, programmed and thoroughly tested first. The country-specific versions were then written by professional translators, automatically back-translated to identify any discrepancies, and fed into the survey software.

The study was run as a cross-sectional survey of adult residents of six EU Member States: Czechia, France, Germany, Hungary, Poland and Romania. We extensively covered Central European countries, seeing larger numbers of displaced people from Ukraine. For the same reason, the number of observations collected in Poland (which hosted by far the largest number of the displaced Ukrainian nationals) scheduled and actually realised exceeded that for other countries, see Table A1.

To maintain representativeness of the general population, hard quotas were set based on Eurostat country statistics for age, gender, education (ISCED), employment status and geographical distribution (NUTS 1) to make sure that they were matched by the sample distribution. In addition, the realisation of soft quotas for the place of residence (urban vs. rural) and income was monitored throughout the data collection process.

Participants were recruited with the use of proprietary and third-party panels associated with a global marketing company, Cint. All panels comprise pre-recruited groups of people who, having been contacted (by any of several ways including direct mailing or calling and the use of website pop-ups), had agreed to participate in online research (surveys/polls/ad. testing). Panel members were contacted by e-mail or push notifications (in the case of members using a mobile app). They received an invitation to the survey; these were sent to all categories of members until a hard quota was met in a given country.

The main fieldwork started Tuesday, August 23 and finished Tuesday, September 6. A soft-launch approach was implemented, allowing for the identification of possible unexpected problems at an early stage. The completion rate, calculated as the fraction of the potential participants contacted in relation to those who actually completed the survey, varied between 79% and 84%, except for Romania, where it was only 43%. Due to this strong selection, combined with the partly delayed realisation of the study as explained in Table A1 note, some caution when looking at the Romanian results is necessary. The average length of the interview ranged from 11:59 in Germany to 13:45 in Czechia.

Final sample size consisted of 15,010 respondents from six countries - the Czech Republic, Germany, France, Hungary, Poland and Romania. The first four countries were represented by a similar number of respondents, while Poland and Romania are overrepresented. The sample was well-balanced in terms of gender, age and education level. There were slight differences in the sample characteristics between the countries, especially regarding the type of residential area, age groups and education. Overall data structure is available in Table 4 and detailed descriptive statistics are available in Appendix A.

**Table 4:** Sample data descriptive statistics.

		Percent	N
Country	Czech Republic	14.1%	2100
	Germany	13.9%	2101
	France	14.1%	2101
	Hungary	13.9%	2101
	Poland	23.4%	3500
	Romania	20.7%	3107
Gender	Female	51.5%	7697
	Male	48.2%	7267
	Other	0.3%	46
Age group	18–25	11.8%	1753
	26–36	17.8%	2657
	36–45	19.4%	2963
	46–55	18.1%	2708
	56–65	16.6%	2450
	65+	16.4%	2479
Residential Area	Rural area	22.5%	4695
	Small city or town	37.4%	3346
	Suburb near a large city	9.0%	5614
	Large city	31.1%	1355
Education	Low (ISCED 1–2)	12.7%	1912
	Medium (ISCED 3–4)	59.6%	8911
	High (ISCED 5–8)	27.7%	4187
Employment Status	Active	59.8%	9035
	Inactive	40.2%	5975

A larger sample was collected in Poland because of the particularly large population of displaced Ukrainians arriving there. In Romania, a translator's mistake was found in the label of one of the levels of one of the attributes of the conjoint experiment as administered to the first 2100 respondents. Moreover, the completion rate was lower than in other countries. A decision to collect additional one thousand observations was made. In practice, no significant differences between the two waves of data collection were found for any variable.

## 6 Weighting and modeling

To correct for the slight deviation of the sample from the quotas (based on the population statistics), we used the SPSS RAKE routine (Kalton and Flores-Cervantes, 2003). It involves a series of small, random changes to the current set of weights until the marginal distributions of the sample and the population are sufficiently similar. This method is generally considered suitable when only marginal distributions in the population are known and there are no reasons to believe that the correlations between the variables are strong. This is indeed the case here, e.g. in the sampled countries, the distribution by education is roughly similar for both genders. For example, in Germany, the largest absolute difference in shares of males and females 25+ attaining a given level of education is just two percentage points: “Fachhochschule or university entrance qualification” is obtained by 36.8% of males and 34.7% of females (microcensus data retrieved from the website of the Statistisches Bundesamt).

As for the econometric strategy, we adopted the multinomial logit model and the mixed logit model to capture the diversity of preferences. This approach enhances our understanding by considering the variations in preferences, offering a more granular view of the factors influencing attitudes towards refugees. The mixed logit framework, with its capability to incorporate random taste variations presents a robust approach to modeling such complex social preferences. Econometric strategy is presented in detail in Appendix C.

This methodology not only refines the accuracy of behavioural predictions but also aids in quantifying the concept of willingness to pay. For any attribute, the WTP, the maximum amount a participant would be willing to pay to switch from one level to another – is simply the estimate for the utility change resulting from such a switch divided by the estimate for the utility of reducing the policy cost by one euro. This definition implies that, for example, if WTP is 10 euro, switching from one level of given attribute to another results in the same loss in utility as the loss of 10 euro would. WTP serves as a gauge for the value placed on policy changes or social interventions. By integrating this into our analyses, we can estimate the economic value that individuals attribute to different attributes associated with policies, as it encapsulates the monetary equivalent of the social and ethical implications surrounding the integration and support of displaced individuals within European societies.

Two alternative approaches to analyse and present data from choice experiments and choice-based conjoint studies are popular in political sciences (Leeper et al., 2020): Average Marginal Component Effects (AMCEs) and Marginal Means (MM).

AMCE can be understood as measuring how much a particular feature's value affects respondents' favorability toward a packaged conjoint profile compared to a baseline (Hainmueller et al., 2014). MM indicates the favorability level toward profiles with a specific feature level, disregarding other features. For instance, in the typical forced-choice design with two alternatives, marginal means directly translate into probabilities (Leeper et al., 2020).

Most conjoint analyses rely on fully randomised designs. However, our study aimed to assess WTP for different programs using the DCE approach. Thus, respondents in our study had the opportunity to opt out (i.e., 'I wouldn't support Temporary Protection in either of these situations'), showing they were not willing to pay the required amount. To estimate AMCE and MM, we thus excluded observations in which option “None” was chosen. Consequently, the DCE design was transformed into the commonly used forced-choice design with two alternatives, as seen in political science research. We report AMCE and MM estimates for completeness and comparability with studies only relying on them.

## 7 Results

The first observation is that participants generally support application of temporary protection in the future. The respondents only choose to grant no protection to any of the groups (option none, the rightmost column in Table 3) in 26% of the cases. This is broadly consistent with previous studies and with the findings from another module of the current study, showing broad support for protection policies for displaced Ukrainian nationals. However, as the situations presented varied in several groups' characteristics, this evidence also suggests that Europeans' support for refugee protection extends to a wide range of situations and people with different characteristics.

### 7.1 Main effects of refugees’ characteristics

We first present the effects of refugees' characteristics on individual attitudes by showing the results for the full sample. We then compare results by treatment. Finally, we study interactions between characteristics of refugees and those of respondents (notably: their nationality). Thanks to the large sample and careful design, the estimates are very precise, yielding clear-cut results, also when zooming in on sub-populations.

**Fig. 1:**
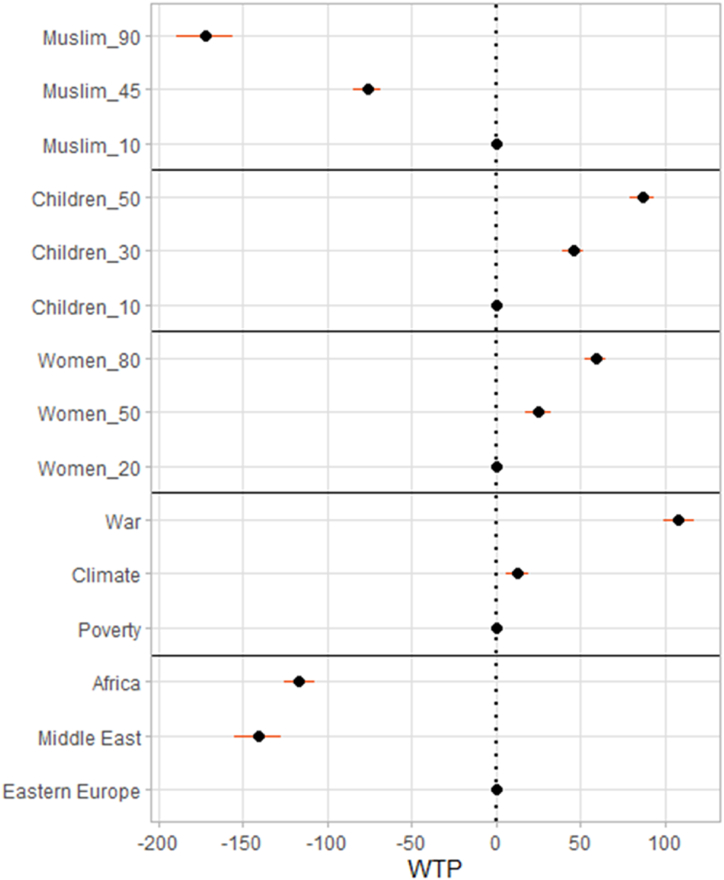
Mean willingness to pay: estimates and 95% confidence intervals (mixed logit regression).

Respondents are more willing to support protecting people displaced by war than economic or climate migrants. As can be seen in Fig. 1, the respondents are willing to pay extra costs of 108.4 euros every year to support people fleeing a military conflict compared to those displaced by adverse economic conditions. Fig. 1 also shows the 95% confidence interval of this estimate, corresponding to the estimated standard error of 4.7 euros (for conciseness henceforth reported as 108.4 ± 4.7). By contrast, the support for protection for climate migrants is only slightly different from that of economic migrants. The additional WTP is just 10.3 ± 3.4 euro, thus nearly 10 times lower than in the case of war refugees. The corresponding AMCEs and MMs are shown in Figures D1 and D2 of Appendix D. For example, other things being equal, being displaced by a war rather than poverty would be expected to increase the probability of being favoured (in a forced choice between two options) by nearly 15 percentage points.

We observe strong and robust effects of age and gender of refugees. Specifically, when the fraction of children among the refugees is high, respondents are ready to pay more for temporary protection. Compared to the baseline of a group with 10% of children, the extra WTP is 40.6 ± 3.2 euro for 30% of children and 86.7 ± 3.8 euro for 50% of children. Alternatively, with costs and all other features unchanged, AMCE would indicate that a group with 30(50)% of children has some 6(11) pct points greater chance of being favoured.

The gender effects on WTP are a bit weaker. Compared to groups with 20% women (the baseline), respondents are willing to pay up to 59.2 ± 3.2 additional euros to support those with 80% women and about 25.0 ± 3.2 extra euros to support gender-balanced groups (50% women). This translates into an effect of about 8 percentage points change in probability of being favoured.

Further, we observe clear, large effects of religious background and country of origin. A higher fraction of Muslims rather than Christians among refugees leads to lower support for protection. Likewise, refugees from the Middle East and Africa are less supported than those from Europe, other things being equal. As shown in Appendix D, these characteristics may change the probability of being favoured by up to some 16 percentage points.

All the main effects are thereby consistent with our pre-registered hypotheses.

### 7.2 Treatments and their interactions

To investigate if our treatment (housing vs. labour vs. control) affected responses, we conducted likelihood-ratio tests comparing the goodness of fit between the baseline model and a model allowing refugee characteristics to have different effects in different treatments. This exercise can be summarised succinctly: we have found no evidence of such differences. The details can be found in Appendix E.

### 7.3 Interactions between characteristics of refugees and respondents

**Fig. 2:**
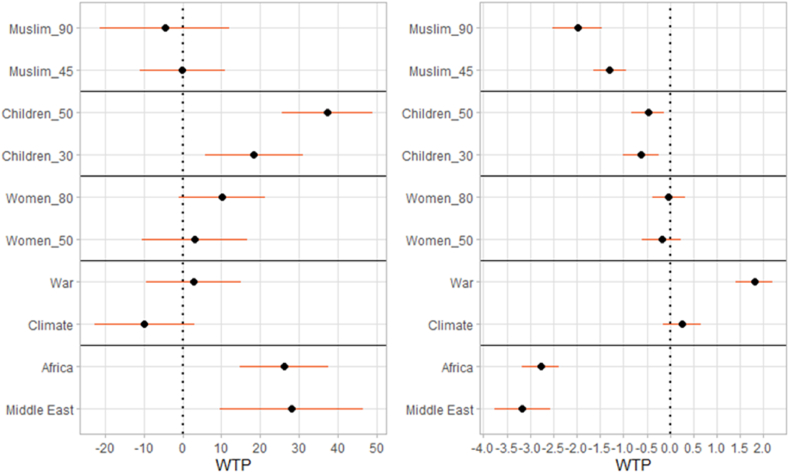
Willingness to pay: interactions with the female gender (left panel) and with age in years (right panel). Estimates and 95% confidence intervals (mixed logit regression). Figure notes: additional WTP, by parameter, among female respondents compared to the male baseline (left panel); additional WTP, by parameter, for each year of age (right panel). Christian_90, Children_10, Women_20, Poverty, Europe as baseline levels.

**Fig. 3:**
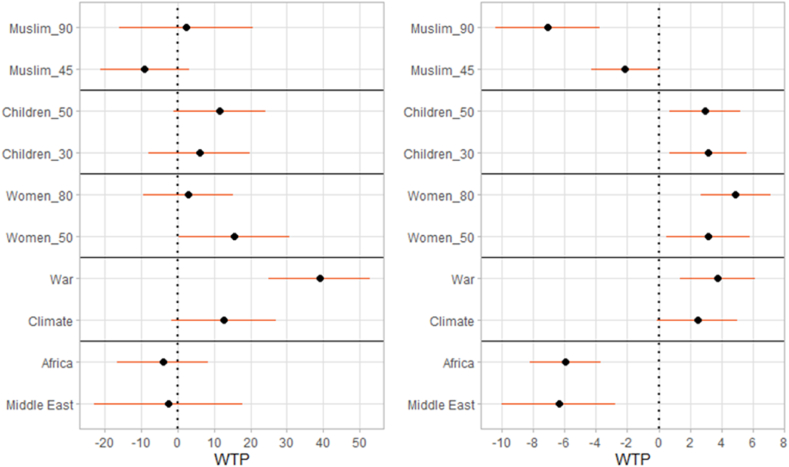
Willingness to pay: interactions with a dummy for higher education (left panel) and with income in euro (right panel). Estimates and 95% confidence intervals (mixed logit regression). Figure notes: additional WTP, by parameter, among respondents with university education vs. base level of no university education (left panel); additional WTP, by parameter, for each euro of monthly income – central points of income brackets assumed (right panel). Christian_90, Children_10, Women_20, Poverty, Europe as baseline levels.

**Fig. 4:**
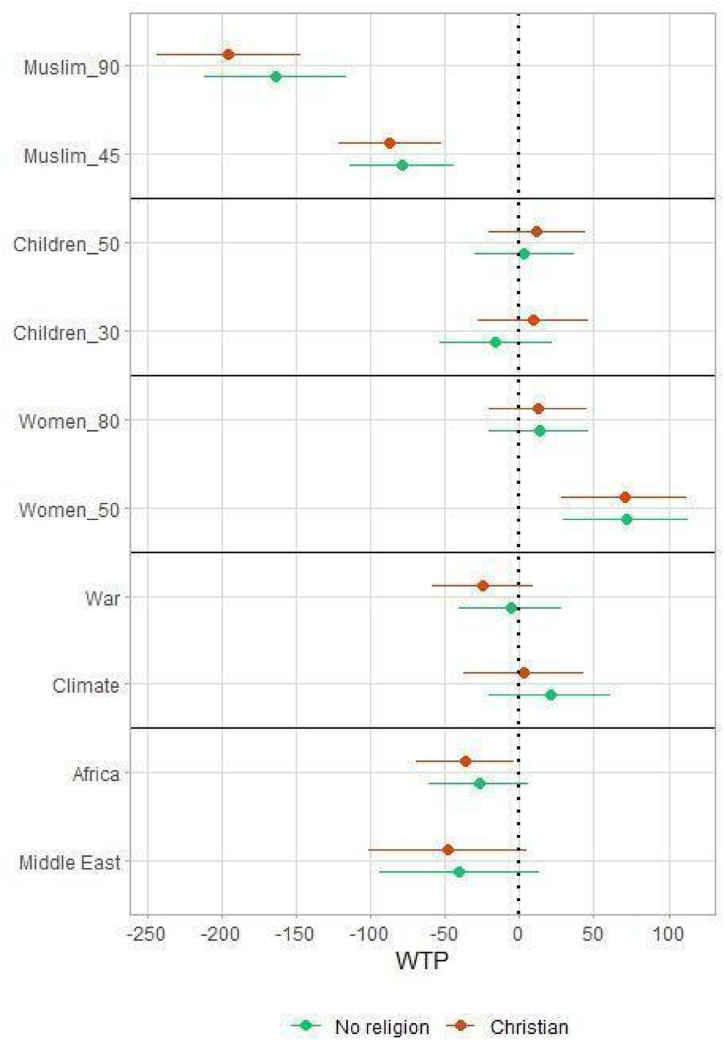
Willingness to pay: interactions with religious denomination. Estimates and 95% confidence intervals (mixed logit regression). Figure notes: additional WTP, by parameter, among respondents declaring to be Christian and those declaring to have no religion, compared to the baseline (Muslim, Jewish and other non-Christian). Christian_90, Children_10, Women_20, Poverty, Europe as baseline levels.

By contrast, we do find evidence of interactions between attributes and demographic characteristics, meaning that the latter are predictive of individual preference, see Fig. 2, Fig. 3, Fig. 4. Again, the corresponding changes in the probability of choosing one option over the other are shown in Appendix D, Figures D3-D5.

First, women are more predisposed to offer protection to minors than men are, see Fig. 2, left panel. For example, compared to men, women are willing to pay an additional 37.3 ± 5.9 euro to support a group composed of 50% rather than 10% of children. This additional effect is more than one-third of the main effect of Children_50 shown in Fig. 1. This corresponds to earlier evidence that, compared to men, women tend to be more sensitive to childrens' calls for help (Messina et al., 2016; Zhang et al., 2022). Moreover, the negative effect of the group of refugees coming from the Middle East or Africa is less pronounced than the one we observe among male responders. Comparing WTPs from Fig. 1, Fig. 2 we see that women's extra WTP is about one-fourth of the main effects.

We also find significant interactions with age. The right panel of Fig. 2 shows that older responders are more negatively predisposed towards Muslims and people from African or Middle-Eastern countries. In particular, one additional year of age corresponds to a drop of 2 euros in the WTP to support groups consisting of 90% Muslims compared to 90% Christians, and the drop is even larger if the refugees are from African or Middle-Eastern countries rather than from European (non-EU) countries. This is consistent with anti-Muslim prejudice being more common among older people (Strabac and Listhaug, 2008), perhaps in part due to limited contact with Muslims in their formative years, although the evidence on age differences in attitudes towards refugees is mixed (Butkus et al., 2016).

Older respondents are also more positively predisposed towards war refugees than economic or climate migrants, with an additional WTP of nearly 1.8 euro for each additional year of age. This effect may be related to being more conscious of war-related atrocities, e.g. due to their parents’ accounts of WWII experiences, although this explanation is somewhat speculative, as we have no direct evidence.

Fig. 3 shows interactions with the respondent's education and income. We find evidence that individuals with university education tend to additionally support people displaced because of war compared to economic migrants, with an additional WTP of 39.0 ± 7.1 euro. We also find a significant interaction for Women_50 (15.6 ± 7.7 euro). Overall, the estimates are less precise than those for the main effects.

Richer individuals generally show higher absolute WTP in most dimensions. Where the main effects are positive (for example, for Children_50), there is an additional positive effect with greater income. Where the main effects are negative (for example, for the Middle East), there is an additional negative effect with greater income. This is so because richer participants have relatively low marginal utility of money; informally, they care a lot about other dimensions, compared to how they care about the cost. This is consistent with our intuitive, pre-registered, hypothesis.

Fig. 4 shows the (limited) effect of religion: the estimates for the two largest groups of respondents, Christians and those declaring no religion tend to be very similar. They both show a negative attitude towards Muslims. This is consistent with homophily because the baseline category encompasses religious but non-Christian respondents and these are mostly Muslim. Christians and non-religious respondents also show higher WTP for groups with more women (although the effect is not linear and only significant for Women_50).

### 7.4 Country-specific effects

**Fig. 5:**
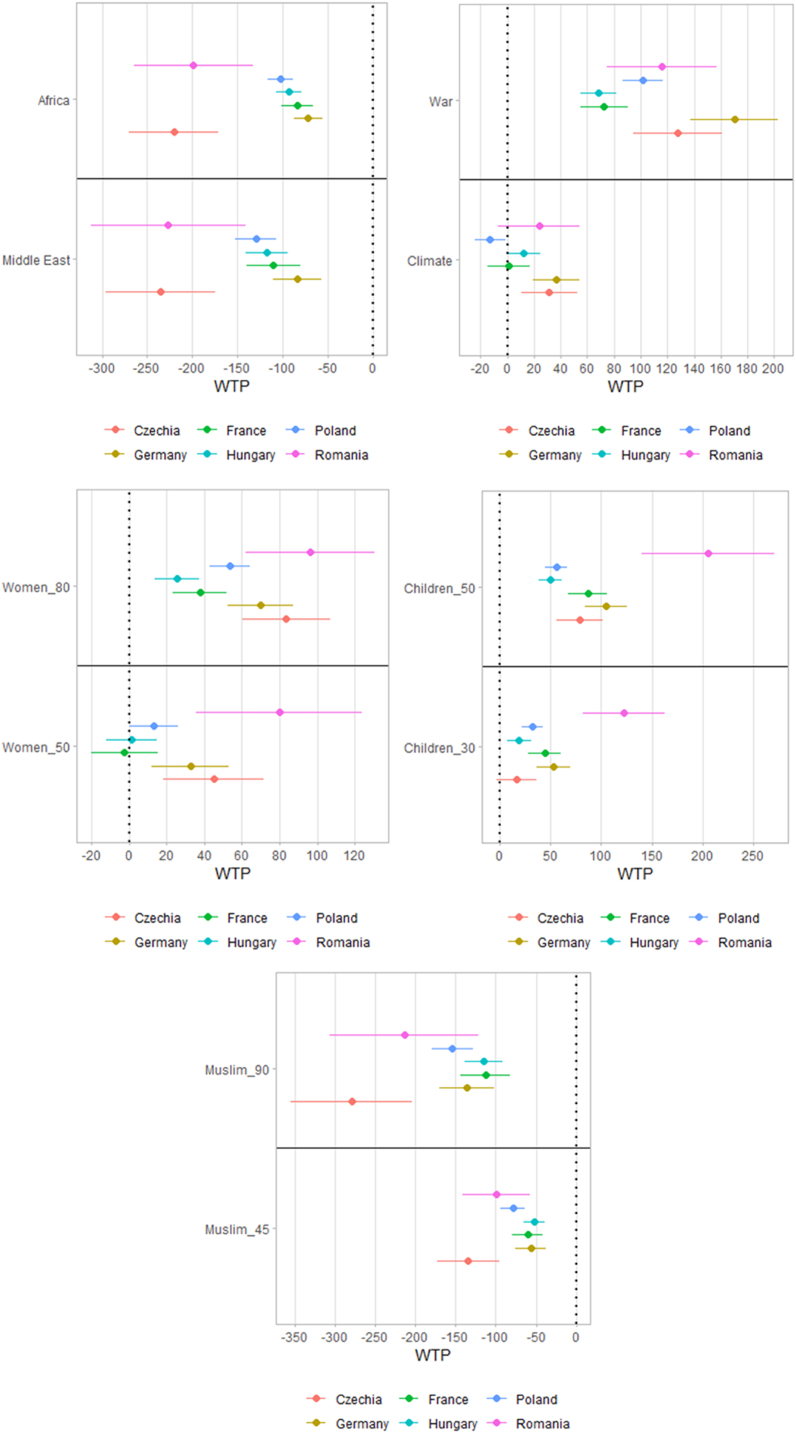
Willingness to pay: differences by countries. Estimates and 95% confidence intervals (mixed logit regression). Figure notes: Christian_90, Children_10, Women_20, Poverty, Europe as baseline levels.

Fig. 5 displays the results of estimations run separately for each country. The effects discussed above are very robust in that they are qualitatively analogous across the six states in our sample. With the only exception of climate migrants in Poland and Women_50 in France (the latter being by far not significant), for each of the 10 parameters, all the six country-specific estimates have the same sign. Quantitatively, however, there are large and telling differences.

In case of both religion and region of origin, the willingness to pay is larger in Czechia and Romania than in the other countries. For example, the additional WTP to support refugees of European origin compared to those from Africa is nearly 200 euro in these two countries compared to 72–102 euro in the other four. Romanians also display very high WTP to support women and children. Germany and Czechia are characterised by particularly high WTP to support migrants displaced by war or climate change. In every country the effect of war (with economic migrants as base category) is several times larger than the effect of climate change (again, with economic migrants as base). Czechia and Romania also tend to have wider confidence intervals, perhaps because a larger fraction of respondents there show nearly-lexicographic preferences, with limited substitution across attributes.

One possible driver of spatial heterogeneity is the distance from Ukraine: many inhabitants of countries and regions close to the Ukrainian borders have recently seen a large number of displaced people arriving or passing through, which may have affected their perception or refugees. To test for this possibility, we have run sensitivity tests, featuring two additional variables: a dummy variable taking the value of one for NUTS 1 regions bordering Ukraine and a continuous variable representing the shortest distance between the region and the Ukrainian border. The results, available upon request, show that the findings reported here are stable when these two distance variables are accounted for, while the two are not significant.

### 7.5 Choices vs. declarations

**Fig. 6:**
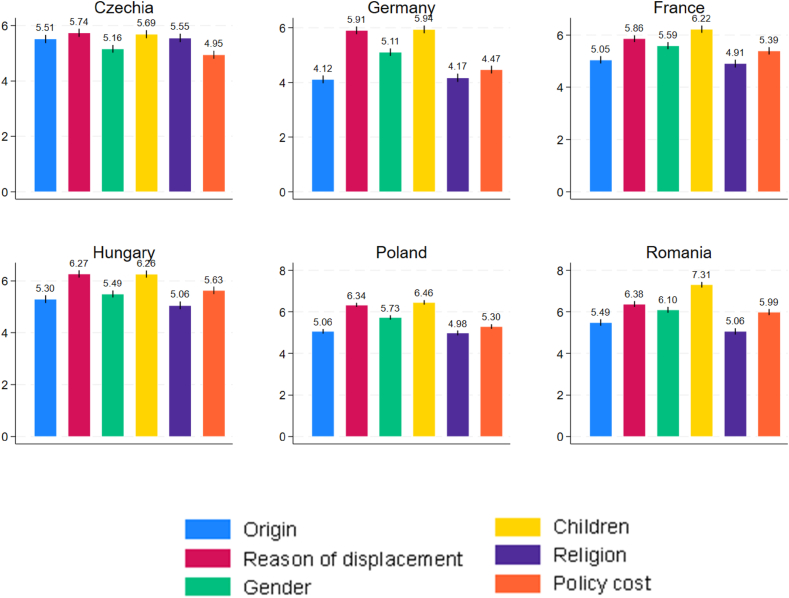
Mean declared importance of attributes [0–10], by country Figure notes: the black vertical lines represent 95% confidence intervals.

We now compare the insights from the conjoint experiment to the respondent's explicit declarations as to how important each attribute (gender, age, displacement motive, etc.) was in shaping their attitudes towards refugee protection. The mean responses to such direct questions, by country and attribute, are shown in Fig. 6. They were rather similar across attributes. For example, in Czechia, the attribute declared to be the least important, namely gender composition of the group of refugees, was rated at 5.2 on the 0–10 scale on average. The most important attribute, “Displaced” (by war vs. climate change vs. poverty) was rated hardly any higher, at 5.8 on average. It should be noted that the low variation did *not* result from people simply mindlessly clicking the same value for all the attributes – such a lack of variance at individual level was only observed in about 7% of respondents. The flat structure of mean declared importance represents a striking contrast with actual importance revealed via conjoint experiment choices: as explored in previous subsections, some attributes tended to make much more of a difference than others did.

Not only is the variation in the rating of the declared importance of the attributes low, but also their ranking tends to be very different from the ranking of the attributes that can be inferred from the choices in the main task. To operationalize the latter, for each country and each attribute, we took the largest estimated WTP for a change from one level to another. For example, in the case of the “Fraction of children” attribute, this would be, for each country, the WTP of a change from 10% to 50% of children. Comparing this WTP to the maximum absolute WTP difference for other attributes we find e.g. that the Fraction of children is more important than the Reason for displacement in Romania but in Poland, the reverse is true. Subsequently, we can calculate, for each country separately, the rank correlation between the two measures of importance of attributes: the one that was thus revealed in choices and the one that was declared (for example, Czechs declaring that “Displaced” was most important and “Fraction of women” was least important). If these orders were identical, the rank correlation would be 1, whereas if they were exactly opposite, it would be −1. It turns out that the Spearman rank correlation coefficient is only relatively high in Germany (0.41), whereas it is actually negative in the remaining five countries, even as low as −0.70 in Poland. This reflects the fact that attributes such as religious background and country of origin tend to be more important than gender composition and fraction of children in actual choices but less important in declarations. In other words, with the exception of Germany, respondents tended to attach much greater actual importance to region and religion and much lower importance to gender composition than they were declaring.

## 8 Discussion and conclusion

In an online conjoint experiment with large samples recruited across six EU countries, we find high willingness to sponsor temporary protection of groups of displaced people with varying religious backgrounds, demographics, and reasons forcing them out of their country. This finding suggests that Europeans' positive attitude toward refugee protection is deeply rooted in their individual preferences and, despite the current war's specific circumstances and the growing weariness, one should expect it to persist over time and in future crises.

However, our study also shows that the willingness to pay for temporary protection varies considerably and systematically across key demographic and socio-cultural characteristics of the group seeking protection. This finding indicates that EU citizens do not view refugees as a homogenous group but instead perceive them as different on several dimensions. For instance, the reasons forcing people to leave their country (war, climate change, economic conditions) significantly influence the willingness to pay for protection. Therefore, by elucidating these differences, our study provides a rigorous way to anticipate or quantify expected changes in public support for protection policies.

As an illustrative example, we discuss how the complexity revealed by our study helps to understand the large difference in EU citizens' attitudes and policy preferences across two cases: the Syrian and the Ukrainians crisis. Many commentators in traditional and social media appeared to imply that this difference can be entirely or almost entirely explained in terms of religious and ethnic differences between these two groups. Our study suggests that differences in the demographic composition and the reasons for displacement have likely played a major role as well. In Table 5, Table 6 and 6, we compare the demographic composition of Syrian asylum applicants in 2015 and the Ukrainians granted temporary protection in 2022. The share of children among the Syrian refugees in 2015 was lower than among Ukrainians in 2022. Even more strikingly, among the adult Syrians applying for asylum in 2015, only one in four was a woman. Among the adult Ukrainians receiving temporary protection in 2022, only one in four was a *man*. Given these differences, our results predict a large difference in attitudes based solely on these demographic differences, irrespective of possible effect of religious or ethnic differences. Given approximate values on all our dimensions, one could calculate that about 80% of respondents would support temporary protection of Ukrainians, compared to just 50% for Syrians. The first number is remarkably close to actual declared support, while for the second we do not have direct evidence although it may be a bit too high in many countries.

**Table 5:** Asylum applicants from Syria in 2015.

	Children	Women 18+	Men 18+
Czechia	45 (33.3%)	40 (29.6%)	50 (37%)
Germany	43,240 (26.6%)	25,595 (15.8%)	93,585 (57.6%)
France	1515 (32.6%)	1315 (28.3%)	1805 (39%)
Hungary	19,380 (30%)	9560 (14.8%)	35,645 (55.2%)
Poland	65 (21.7%)	75 (25%)	155 (51.7%)
Romania	160 (29.1%)	110 (20%)	155 (50.9%)
EU total	106,170 (29.5%)	62,210 (17.3%)	191,545 (53.2%)

Retrieved Dec. 11, 2022 from Eurostat's Asylum applicants by type of applicant, citizenship, age and sex - annual aggregated data. Row percentages in parentheses.

**Table 6:** Decisions granting Temporary Protection to displaced Ukrainian nationals, March–September 2022.

	Children	Women 18+	Men 18+
Czechia	139,670 (32.8%)	201,150 (47.2%)	84,920 (19.9%)
Germany	214,100 (32.9%)	324,430 (49.9%)	110,305 (17%)
Poland	645,525 (45%)	678,325 (47.3%)	111,170 (7.7%)
Romania	25,165 (35.7%)	31,340 (44.5%)	13,960 (19.8%)

Retrieved Dec. 11, 2022 from Eurostat's Decisions granting temporary protection by citizenship, age and sex – quarterly data. Data for France, Hungary and the EU as a whole is missing. Row percentages in parentheses.

Our findings are also broadly in line with earlier studies focusing on individual asylum applicants, suggesting robustness of conjoint experimentation applied to attitudes towards refugees. One difference that we note is that our study showed relatively strong effects of the region of origin, for example when compared to those reported by Bansak et al. (2016) (although caution is required given other methodological differences between the two studies). While not hypothesised, this seems a natural effect of our design choice to ask the participants to compare whole groups: a collective notion of nationality is likely to affect perception of the group to a greater extent than perception of a single individual.

Our study suggests that EU citizens nearly equally support temporary protection of people forced to leave their country because of adverse climate change and those migrating for economic reasons (while the third condition – being displaced by a war – triggers very different reactions). One plausible reason for the result is that climate change is generally perceived as a gradual process, making it difficult to distinguish (hypothetical) climate migration from other types of migration. It should be emphasised that our framing used general, abstract terms; it is plausible that displacement caused by more specific and salient events which can be partly blamed on climate change (such as a major flood or draught) would be met with more willingness to trigger protection.

Our study shows substantial variation in preferences across countries and different segments of the population within each country. While the effects are qualitatively analogous for all countries, their sizes vary greatly. Our estimates can thus be used to quantify and anticipate social tensions across European communities dealing with new refugee crises. Even so, temporal stability of these differences cannot be taken for granted, calling for periodic re-runs of such a design, ideally in a broader group of countries.

Additionally, within the geographic and thematic scope of our study, we find no evidence of an effect of communication concerning specific aspects of temporary protection, such as whether the beneficiaries of temporary protection can enjoy access to public housing or the labour market, on the average levels of support for temporary protection. Indeed, our treatments yielded about the same level of support on average.

By contrast, our findings suggest that information campaigns and public messages concerning demographic composition of migration flows can effectively influence public opinion. There is limited evidence as to whether the European public opinion holds a correct understanding of the demographics of the incoming populations. However, in another module of our study, we find that participants tend to underestimate the fraction of women among displaced Ukrainian nationals. In view of our conjoint experiment results, correcting these beliefs could make a positive impact on the attitude toward protection of displaced Ukrainians.

It is also important to point out that, while many characteristics of refugee groups are fixed, other traits could be affected by targeted migration policy. For example, using regression discontinuity design, Arendt et al. (2020) showed that language training improved refugees’ labour market outcomes in Denmark. Orientation in various important domains such as parenting and healthcare, recognition of job market qualifications etc. can be addressed in orientation programs; research conducted in Germany (Weiss and Tulin, 2021) showed that mere participation in such programmes facilitated acceptance of its beneficiaries by the local population. At the same time, such interventions will only have a limited effect. One should acknowledge the results of our study and many others: that immutable characteristics of migrants (such as gender and country of origin) make a big difference for the way they are perceived in the host country, with obvious consequences for their chance to integrate and thrive.

To be sure, our study does not account for all possible drivers of preference, some of which may be correlated with the dimensions we manipulated. For example, an early reader suggested that refugees from Eastern Europe may be perceived to be more likely to return (soon) to their country of origin, compared to those coming from more distant locations. This could partly explain preference for this group. As we explicitly asked our participants to consider a one-year period (and we asked them to focus on several other – varying – characteristics instead), we do not think this was a very common consideration. Relatedly, we do not have comments from participants that would suggest they inferred a difference in the expected length of stay (and that it affected their choices). Still, it could be an interesting dimension to consider in future research.

Another reader suggested that the scenario of climate change displacing (a large group of) Eastern Europeans was less believable. It is a concern, although none of the comments we received during design, testing and data collection hinted at that. This is why we could not have excluded this combination by design, nor have we inquired if indeed participants found it less plausible. As second-best, we run robustness tests by eliminating, ex-post, all choice sets containing this particular combination. It turned out to make no qualitative differences, although the estimate for Climate Change raised slightly. In future studies, it seems desirable to consider excluding such a combination by design.

By contrast, we have eliminated the possibility of a predominantly Muslim group from Eastern Europe and a predominantly Christian group from the Middle East by design. This was because the seemingly common misnomer of identifying Muslims with Arabs would make such scenarios less believable. We cannot be completely sure whether including them could change the results. Again, a possible verification is left for future scholars.

Another limitation of our study, common to nearly all conjoint experiments, is that respondents are confronted with hypothetical questions. This may cause external validity concerns: to what extent do the findings generalise to choices with real consequences? This issue was carefully examined by Hainmueller et al. (2015). They found that the results of conjoint and vignette experiments matched the results of corresponding referendums in Switzerland “remarkably well”; while design choices made a difference, paired conjoint designs (such as ours) performed the best. One feature of this method is worth emphasising in this context: it makes it less transparent to the participants that their preferences concerning specific attributes may be inferred. Thus, even if they are hesitant to explicitly admit to some preferences (for example because they recognise them to violate social norms) they are unlikely to make an effort to hide them in the choice-based conjoint experiment. This is consistent with our observation that the importance ratings explicitly assigned to different dimensions were very different from the weights of these dimensions as revealed in the main choice task. Naturally, validation in a consequential task (such as a referendum) remains an attractive option for future research.

## Funding

We are very grateful to Tim Hatton, our colleagues at CCBI JRC, as well as participants of workshops and conferences in Brussels, Florence, Santa Barbara and Warsaw. Remaining errors and omissions are ours. This study was funded by the 10.13039/501100000780European Commission. The contents of this publication do not necessarily reflect the position or opinion of the European Commission. M. Giergiczny acknowledges the financial support from the National Science Centre in Poland (2021/43/B/HS4/03371).

## CRediT authorship contribution statement

**Michal Krawczyk:** Writing – review & editing, Writing – original draft, Project administration, Methodology, Investigation, Conceptualization. **Andrea Blasco:** Writing – review & editing, Methodology, Data curation, Conceptualization. **Tomasz Gajderowicz:** Writing – review & editing, Visualization, Software, Methodology, Data curation. **Marek Giergiczny:** Writing – review & editing, Visualization, Software, Data curation.

## Declaration of competing interest

The authors declare that they have no financial or personal interests or beliefs that could affect their objectivity.

## Data Availability

Data will be made available on request.
