# Supplementary material for: Support for temporary protection of displaced populations in the EU: A conjoint experiment
Source: Eur J Polit Econ. 2024 Dec;85:None. doi: 10.1016/j.ejpoleco.2024.102601 (PMC11649312; doi:10.1016/j.ejpoleco.2024.102601)
Supplement: Multimedia component [file mmc1.docx]

Appendix A: Descriptive statistics

Table A1. Sample data descriptive statistics by country

|  |  | Czech Republic | | Germany | | | France | | | Hungary | | | Poland | | | Romania | | |  |
| --- | --- | --- | --- | --- | --- | --- | --- | --- | --- | --- | --- | --- | --- | --- | --- | --- | --- | --- | --- |
|  |  | Percent | N | | Percent | N | | Percent | N | | Percent | N | | Percent | N | | Percent | N | |
| Gender | Female | 51.8% | 1026 | | 51.4% | 1027 | | 52.4% | 1000 | | 52.0% | 994 | | 51.9% | 1672 | | 49.8% | 1548 | |
|  | Male | 48.0% | 1069 | | 48.3% | 1067 | | 47.5% | 1098 | | 47.7% | 1102 | | 47.5% | 1811 | | 49.8% | 1550 | |
|  | Other | 0.2% | 5 | | 0.3% | 7 | | 0.1% | 3 | | 0.3% | 5 | | 0.6% | 17 | | 0.4% | 9 | |
| Age group | 18 - 25 | 9.9% | 211 | | 10.9% | 230 | | 12.6% | 272 | | 11.6% | 242 | | 11.4% | 392 | | 13.5% | 406 | |
|  | 26 - 36 | 16.5% | 363 | | 17.3% | 365 | | 16.3% | 346 | | 16.9% | 347 | | 19.4% | 656 | | 18.9% | 580 | |
|  | 36 - 45 | 20.7% | 438 | | 16.6% | 337 | | 18.4% | 372 | | 19.6% | 421 | | 20.3% | 743 | | 20.0% | 652 | |
|  | 46 - 55 | 18.1% | 369 | | 17.1% | 359 | | 16.4% | 357 | | 19.1% | 408 | | 16.5% | 574 | | 20.8% | 641 | |
|  | 56 - 65 | 15.0% | 314 | | 17.8% | 373 | | 16.5% | 335 | | 17.6% | 349 | | 17.8% | 604 | | 15.1% | 475 | |
|  | 65+ | 19.9% | 405 | | 20.5% | 437 | | 19.8% | 419 | | 15.2% | 334 | | 14.6% | 531 | | 11.6% | 353 | |
| Residential Area | Rural area | 24.4% | 505 | | 17.8% | 382 | | 28.4% | 613 | | 23.1% | 480 | | 20.6% | 706 | | 21.9% | 660 | |
|  | Small city or town | 44.7% | 933 | | 40.8% | 857 | | 36.2% | 756 | | 41.5% | 870 | | 37.7% | 1324 | | 27.9% | 874 | |
|  | Suburb near a large city | 5.9% | 127 | | 16.7% | 349 | | 16.7% | 352 | | 6.0% | 131 | | 6.6% | 228 | | 5.7% | 168 | |
|  | Large city | 25.0% | 535 | | 24.7% | 513 | | 18.7% | 380 | | 29.5% | 620 | | 35.2% | 1242 | | 44.6% | 1405 | |
| Education | Low (ISCED 1-2) | 7.9% | 166 | | 18.4% | 395 | | 15.7% | 337 | | 12.3% | 247 | | 10.9% | 380 | | 12.6% | 387 | |
|  | Medium (ISCED 3-4) | 69.6% | 1454 | | 52.5% | 1093 | | 45.1% | 941 | | 60.2% | 1283 | | 59.5% | 2062 | | 66.9% | 2078 | |
|  | High (ISCED 5-8) | 22.5% | 480 | | 29.1% | 613 | | 39.2% | 823 | | 27.5% | 571 | | 29.6% | 1058 | | 20.5% | 642 | |
| Employment Status | Active | 62.1% | 1560 | | 62.3% | 1584 | | 57.8% | 1730 | | 62.6% | 1562 | | 60.2% | 2784 | | 55.4% | 2730 | |
|  | Inactive | 37.9% | 1320 | | 37.4% | 1309 | | 42.2% | 1236 | | 37.4% | 1320 | | 39.4% | 2108 | | 44.6% | 1742 | |

Appendix B: Survey questionnaire

[all instances of “Germany” below replaced with relevant country in other versions; euro amounts converted into local currencies using PPP];

Welcome!
Thank you for contributing to this study. It is being conducted by VVA Brussels for the Joint Research Centre of the European Commission. The study intends to provide the European Commission with an objective picture of the opinions and attitudes of EU citizens towards policies that protect people who are forced to leave their country and arrive in the EU.
The information you provide will be treated as confidential in accordance with the GDPR (EU) 2018/1725 and will only be shared in an anonymized format with the Joint Research Centre of the European Commission. In the study, your responses will be combined with those of many others and summarised in aggregate values to further protect your anonymity.

Every year, events such as wars and natural disasters force people to leave their homes and flee their country to save their lives. The survival of these people depends on international assistance and protection. The objective of this survey is to better understand Europeans’ views on the current and future policies to support displaced people that seek shelter in the European Union.

Public opinion surveys such as this one may shape policy changes. Therefore, it is important that you read each question carefully and respond in line with your personal beliefs and preferences.

Q1 What is your age?

______________________________

Q2 What is your gender?

1. Male

2. Female

3. Other

Q3 Which term best describes the place you live in?

1. Rural area

2. Small city or town

3. Suburb near a large city

4. Large city

Q4 In which region do you live?

[country-specific menu of responses]

Q5 What is the highest academic degree achieved?
[country-specific menu of responses]

Q6 Which of these descriptions best describes your situation in the last seven days?

1. In paid work

2. In education

3. Unemployed and actively looking for a job

4. Unemployed, wanting a job, but not actively looking for a job

5. Permanently sick or disabled

6. Retired

7. In community or military service

8. Doing housework, looking after children or other persons

9. Other

We would like you to compare different groups of people that are forced to leave their country and arrive in the EU for different reasons.
Assume that the group size is such that there would be 1 displaced person per 500 residents of Germany.
These groups may differ in terms of the following characteristics:

| Country | Country of origin |
| --- | --- |
| Displaced by | The reason for which they had to flee their country of origin |
| Fraction of women | The fraction (percentage) of women among the displaced adults |
| Fraction of children | The fraction (percentage) of children below the age of 14 |
| Religious background | Religious background |
| Yearly cost for you | This is the yearly cost, to you, of the policy of temporary protection of this group of displaced persons. Every policy has its costs that are generally covered with taxes that you and other citizens pay (fiscal costs). |

Q7_1 Which of group A and B, if any, would you offer temporary protection in the EU? Here, temporary protection means they will receive…

1. … a residence permit, medical care, and obtain housing in Germany for a year. [Housing Treatment]

2. … a residence permit, medical care, and access to the labour market in Germany for a year. [Labour Treatment]

3. … a residence permit and medical care in Germany for a year. [Control Treatment]

Scenario 1 of 6

| Attribute | Situation A | Situation B | None |
| --- | --- | --- | --- |
| Country of origin | Sub-Saharan African country such as Nigeria or Ethiopia | A Middle Eastern country such as Syria or Lebanon | I wouldn’t support  Temporary  Protection in any of  these two situations |
| Displaced by | A war in their country | Poverty in their country |  |
| Fraction of women | 50 out of 100 adults are women | 20 out of 100 adults are women |  |
| Fraction of children | 10 out of 100 are children | 30 out of 100 are children |  |
| Religion | 45 out of 100 are Christian, 45 are Muslim | 90 out of 100 are Muslim |  |

Q8 You were asked to make choices between options with six characteristics. To what extent did you consider each of these characteristics when making your choices? Please use the sliders to indicate how important each characteristic was, ranging from 0 -- not at all important to 10 -- extremely important.

|  | 0 | 1 | 2 | 3 | 4 | 5 | 6 | 7 | 8 | 9 | 10 |
| --- | --- | --- | --- | --- | --- | --- | --- | --- | --- | --- | --- |
| Country of origin |  |  |  |  |  |  |  |  |  |  |  |
| Displaced by: |  |  |  |  |  |  |  |  |  |  |  |
| Fraction of women: |  |  |  |  |  |  |  |  |  |  |  |
| Fraction of children: |  |  |  |  |  |  |  |  |  |  |  |
| Religious background: |  |  |  |  |  |  |  |  |  |  |  |
| Yearly cost per EU citizen: |  |  |  |  |  |  |  |  |  |  |  |

We now ask you to consider the current situation of people leaving Ukraine because of the war and arriving in Germany.

[one out of three versions of Q9 shown:]

Q9A Imagine fleeing your home with little more than the clothes you are wearing and the few items you can carry. You are now in a foreign land and must find a way to obtain money to pay for food and other basic needs. Could you describe what you would do to survive and what would be the biggest challenge for you in this situation?

____________________________________________________________

____________________________________________________________

Q9B A two-year-old boy's mother arrived from Ukraine with her child soon after the war began. She has been recently interviewed about her situation: “Our fridge is empty, so we need to buy food,” she said. “Some people have tried to give us food, but I feel ashamed. I want to buy it myself.” Could you describe your feelings in hearing about this situation?

____________________________________________________________

____________________________________________________________

Q9C Could you describe how do you stay informed about the recent events in the war in Ukraine (news, social media, friends, ...)?

____________________________________________________________

____________________________________________________________

Q10 Do you agree or disagree with granting displaced people from Ukraine temporary work permits in Germany?

|  | 0 | 1 | 2 | 3 | 4 | 5 | 6 | 7 | 8 | 9 | 10 |
| --- | --- | --- | --- | --- | --- | --- | --- | --- | --- | --- | --- |
| LOA temporary work permits |  |  |  |  |  |  |  |  |  |  |  |

Q11 Some people have donated money to help Ukrainians cover their most immediate basic needs. Would you agree to write a short text to persuade people in to donate?

We will share your message on our Twitter account @ukrnSupportSurvey.

1. Yes

2. No

Please enter here the text of your message (You can also edit our template); @ukrnSupportSurvey

Remaining characters: 280 ____________________________________________________________ ____________________________________________________________

Q12 Over the last months, some locals helped displaced people from Ukraine staying in Germany in various ways, others did not want to do that or did not have such a possibility. Have you, in the last four months, been helping people from Ukraine, in any of the following ways?

Click all that apply

1. By donating money

2. By donating goods (clothes, food, medicines, …)

3. By taking part in some volunteering activity

4. By hosting refugees from Ukraine

5. By supporting refugees from Ukraine on social media

6. I was helping in another way

7. I was not actively helping in the past four months

Q13 Do you have family members or close friends who are helping people from Ukraine?

1. Yes

2. No

Q14 On March 4, 2022, the European Council approved broad temporary protection to offer quick and effective assistance for people fleeing the war in Ukraine. It gives them the right to reside and travel across the EU, access to education, medical care, employment, and accommodation for a period of one year. Do you support or do you oppose this decision?

1. [from 0 – I oppose it very strongly – to 10 – I support it very strongly]
2. I don't know

Q15 Please, indicate to what extent you agree or disagree with each of the following statements about temporary protection in the EU:

|  | Strongly disagree | Disagree | Neither agree or disagree | Disagree | Strongly disagree |
| --- | --- | --- | --- | --- | --- |
| … it should offer housing rights (social housing, controlled rent, etc.) |  |  |  |  |  |
| … it should offer access to the labour market (work permits, etc.) … |  |  |  |  |  |
| … it should offer access to public education |  |  |  |  |  |
| … it should offer access to medical care |  |  |  |  |  |
| … it should offer the right to move freely within the EU |  |  |  |  |  |

Q16 This protection will expire on March 23, 2023; one year after it was triggered. Assuming the situation in Ukraine does not change much, do you think the EU should extend the temporary protection for Ukrainian people beyond March 23, 2023?

1. No, we should not extend it

2. Yes, we should extend it for one more year

3. Yes, we should extend it for at least two more years

4. I don’t know

Q17 Who do you think is responsible for the war in Ukraine?

Click one or more parties that you think are at least partly responsible

1. Ukrainian authorities

2. Russian authorities

3. US authorities

4. EU authorities

5. Another government or organization

6. I don’t know/I don’t want to respond

Q18 To the best of your knowledge, how many people from Ukraine displaced by the war are currently in Germany?

1. Less than 10 thousand

2. 10-100 thousand

3. 100-300 thousand

4. 300-500 thousand

5. 500 thousand – 1 million

6. 1 – 2 million

7. 2 – 4 million

8. 4 – 6 million

9. 6 – 8 million

10. More than 8 million

Q19 What costs does Germany incur by providing Ukrainian refugees with medical care, food, etc.? In your opinion, what is the average cost per day of a refugee's stay?

1. less than 20€
2. 21-40€
3. 41-60€
4. 61-100€
5. 101-300€
6. More than 300€

Q20 If a person coming from Ukraine is religious, what would be your best guess about the denomination?

1. Christian

2. Jewish

3. Muslim

4. Another religion

5. I don’t know

Q21 To the best of your judgement, out of every 100 people from Ukraine who have arrived in Germany because of the war, how many are...

Please note that the three numbers should add up to 100

|  | Percentage |
| --- | --- |
| Children below 14 years old? |  |
| People aged 14-60? |  |
| Seniors above 60 years old? |  |

Q22 How many of the adult Ukrainians who have arrived in Germany...

| Are women? |  |
| --- | --- |
| Are native speakers of Ukrainian? |  |
| Are native speakers of Russian? |  |
| Can communicate in English? |  |
| Can communicate in German? |  |
| Have a higher education? |  |

Q23 To what extent do you agree or disagree with the statement: “Ukraine is part of the EU family”?

|  | 0 | 1 | 2 | 3 | 4 | 5 | 6 | 7 | 8 | 9 | 10 |
| --- | --- | --- | --- | --- | --- | --- | --- | --- | --- | --- | --- |
| UA_EU_family |  |  |  |  |  |  |  |  |  |  |  |

Q24 In the last 4 weeks, have you interacted with people from Ukraine?

Interaction can mean anything from exchanging a few words to doing an activity together

1. Yes, every day or almost every day

2. Yes, at least once a week

3. Yes, occasionally

4. Never

Q25 And before the start of the war, had you interacted with people from Ukraine?

1. Yes, every day or almost every day

2. Yes, at least once a week

3. Yes, occasionally

4. Never

Q26 Regarding immigration in general, would you say it is good or bad for Germany’s economy that people come to live here from other countries?

|  | 0 | 1 | 2 | 3 | 4 | 5 | 6 | 7 | 8 | 9 | 10 |
| --- | --- | --- | --- | --- | --- | --- | --- | --- | --- | --- | --- |
| immigration_good |  |  |  |  |  |  |  |  |  |  |  |

Q27 In politics, people sometimes talk about “left” and “right.” Where would you place yourself on this scale, where 0 means the left and 10 means the right?

|  | 0 | 1 | 2 | 3 | 4 | 5 | 6 | 7 | 8 | 9 | 10 |
| --- | --- | --- | --- | --- | --- | --- | --- | --- | --- | --- | --- |
| political_right |  |  |  |  |  |  |  |  |  |  |  |

Q28 Should Germany’s government promote programs to reduce income differences between the rich and poor?

|  | 0 | 1 | 2 | 3 | 4 | 5 | 6 | 7 | 8 | 9 | 10 |
| --- | --- | --- | --- | --- | --- | --- | --- | --- | --- | --- | --- |
| reduce_gap |  |  |  |  |  |  |  |  |  |  |  |

Q29 Would you say that “most people can be trusted” or do you believe that “you can’t be too careful when dealing with people”?

|  | 0 | 1 | 2 | 3 | 4 | 5 | 6 | 7 | 8 | 9 | 10 |
| --- | --- | --- | --- | --- | --- | --- | --- | --- | --- | --- | --- |
| trust |  |  |  |  |  |  |  |  |  |  |  |

Q30 Do you consider yourself as belonging to any religion?

1. Roman Catholic

2. Protestant

3. Eastern Orthodox

4. Other Christian denomination

5. Jewish

6. Islamic

7. Other non-Christian religions

8. I do not belong to any religion

Q31 Regardless of whether you belong to a particular religion, how religious would you say you are?

|  | 0 | 1 | 2 | 3 | 4 | 5 | 6 | 7 | 8 | 9 | 10 |
| --- | --- | --- | --- | --- | --- | --- | --- | --- | --- | --- | --- |
| religion_intensity |  |  |  |  |  |  |  |  |  |  |  |

Q32 What is your current marital status?

1. I am married

2. I am not married but I have a partner, and we live together

3. I have a partner, but we do not live together

4. I am single

Q33 How many children do you have, if any?

1. Number of children: ____________________

2. I don’t have any children

Q34 In which country were you born?

Q35 Were any of your parents born in a different country?

1. Yes, both of them

2. Yes, only my father

3. Yes, only my mother

4. No, none of them

Q36 In which country was your father born?

Q37 In which country was your mother born?

Q38 Which level describes your monthly gross pay?

[country-specific brackets]

This question completes the survey.

Many thanks for providing your input!

Appendix C: Econometric strategy

The theories of economic value (Lancaster, 1966) and random utility theory (McFadden, 1974; McFadden, 2001) provide the foundations for modelling preferences using discrete choice. Typically, discrete choice models are derived with the assumption that the decision-maker maximises utility. The resulting utility is decomposed into the individual utility associated with observed characteristics of a particular option and unobserved ones, represented by a stochastic component. The expression of the utility of an individual, $i$, resulting from choosing an alternative, $j$, in situation, $t$, is as follows:

$$U_{ijt}=X_{ijt}\beta+e_{ijt} (1)$$

The (1) expression is separable in the observed choice attributes, $X_{ijt}$, with the vector of parameters $\beta,$ and $e_{ijt}$ – the stochastic component accounting for factors not observed during analysis. If it is assumed that the stochastic component ($e_{ijt}$) follows an independent and identical extreme value (type I) distribution, then it leads to a familiar logit probability specification, as used in simple conditional logistic regressions:

$$P\left( j | J \right)=\frac{exp\left( X_{ijt}\beta\right)}{\sum_{k=1}^{J} exp\left( X_{ikt}\beta\right)} (2)$$

which can then be used to derive the maximum likelihood estimator of the utility function parameters, given the observed choices of individuals and the attribute levels associated with choice alternatives. The formula in (2) is the multinomial logit model (MNL) which can be used to derive the maximum likelihood of the utility function parameters, given the observed choices and attribute levels associated with choice alternatives of the individuals.

Despite the practicality of logit models in representing choice behaviour, their application has several limitations. MNL can only represent taste variation that is associated with the observed characteristics of the decision-maker (Train and Weeks, 2003). The model can represent so-called systematic taste variation but not random taste variation which can be conceptualised as differences in tastes that cannot be linked to observable characteristics. The second limitation is that logit cannot account for unobserved factor correlations over time.

To relax these assumptions and allow for heterogeneity of preferences, the individual-specific parameters, $\beta$, have to be included. The relaxation of these assumptions leads to more generic models. If individual parameters are assumed to be continuously distributed following a parametric distribution specified a priori by the modeller, $\beta_{i}\sim f\left( b,\Sigma\right)$, with means ***b***, and variance-covariance matrix, $\Sigma$, the random parameters mixed logit model is formed (McFadden and Train, 2000).

In addition to the MNL, we employed the mixed logit model (MIXL). The expressions for mixed logit probabilities are the integrals of standard logit probabilities over a density of parameters.

$$P_{ni}=\int\frac{e^{\beta_{n}^{'}x_{ni}}}{\sum_{j} e^{\beta_{n}^{'}x_{nj}}}\phi\left( \beta|b,\Omega\right)d\beta(3)$$

For the MIXL, we used the panel specification as proposed by Revelt and Train (1998). The parameters (β_n_) vary across respondents but stay constant across choice for the same respondents.^^[[1]](#footnote-1)^^ In our work, we assumed a mixture of normal and log-normal density with mean b and unrestricted covariance Ω.

By assumption, all non-price coefficients follow a normal distribution and the price coefficient follows a log-normal distribution. From a behavioural perspective, the assumption of the lognormal distribution for the price coefficient is plausible: it requires all respondents to have negative price sensitivity. This assumption is the standard practice in WTP-space models. It prevents simulated cost parameters to be close to zero, and thus guarantees that the resulting distributions of Willingness to Pay (WTP) have finite moments (Daly et al., 2012). For any attribute, we can calculate the maximum amount a participant would be willing to pay to switch from one level to another – it is simply the estimate for the utility change resulting from such a switch divided by the estimate for the utility of reducing the policy cost by one euro.

Appendix D: AMCE and MM analysis


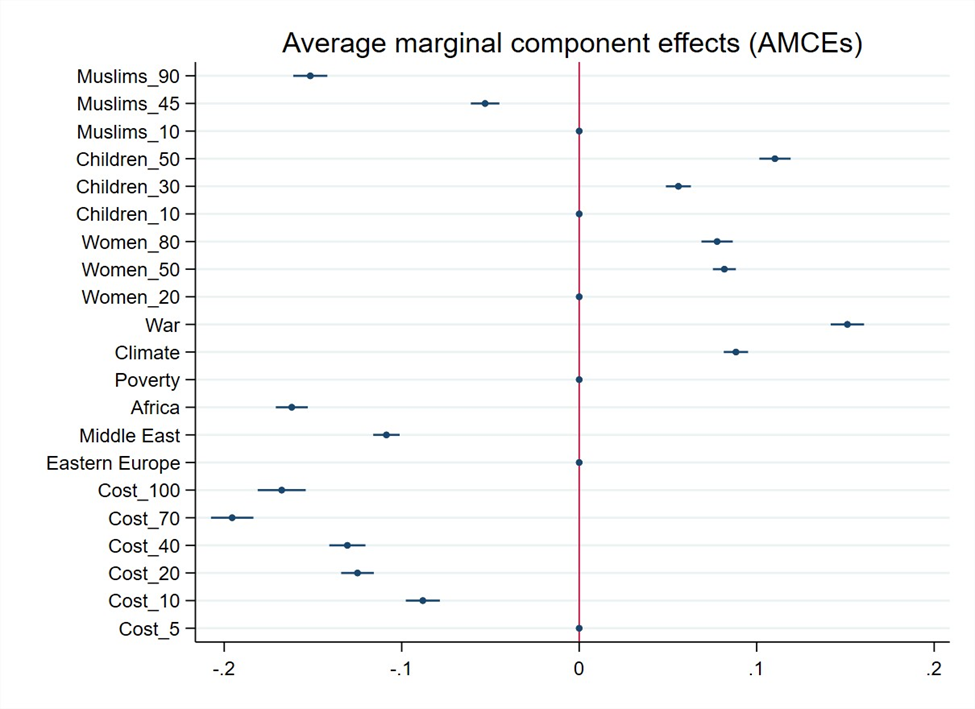


Figure D1. AMCE with 95% confidence intervals


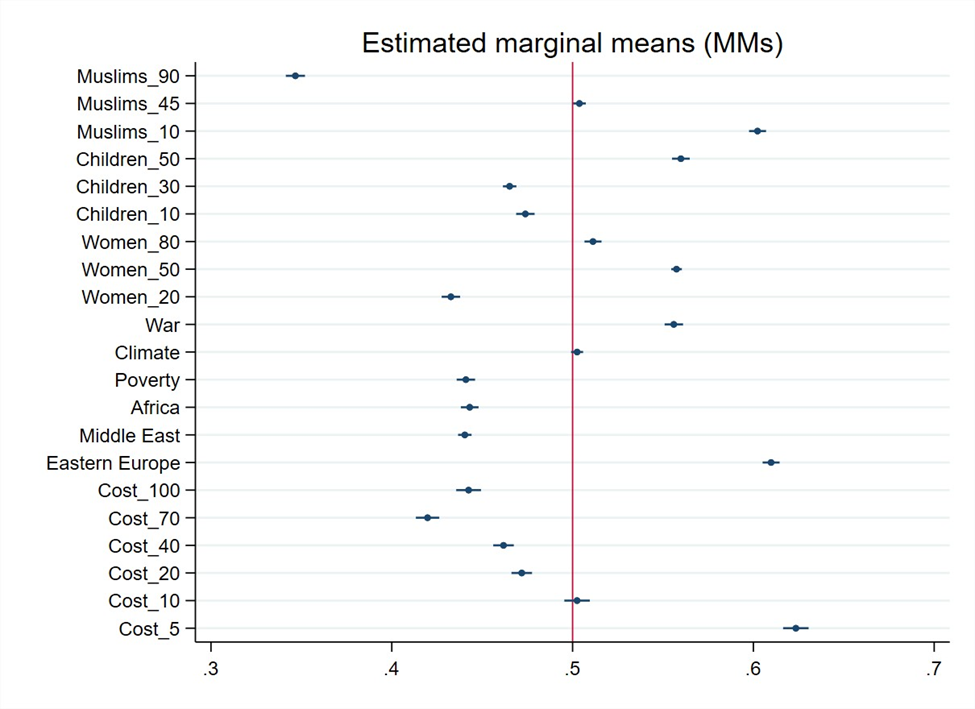


Figure D2. MMS with 95% confidence intervals


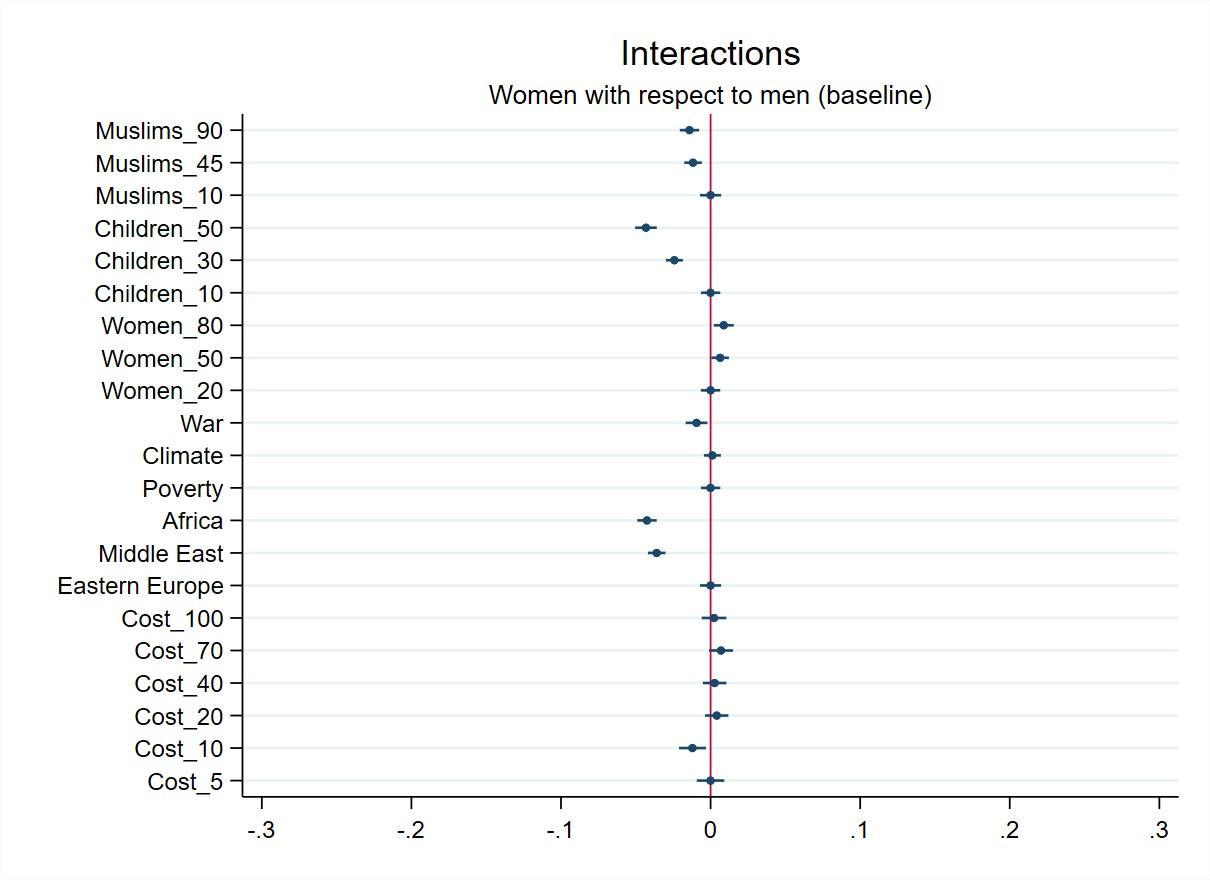


Figure D3. AMCE with 95% confidence intervals for gender interactions


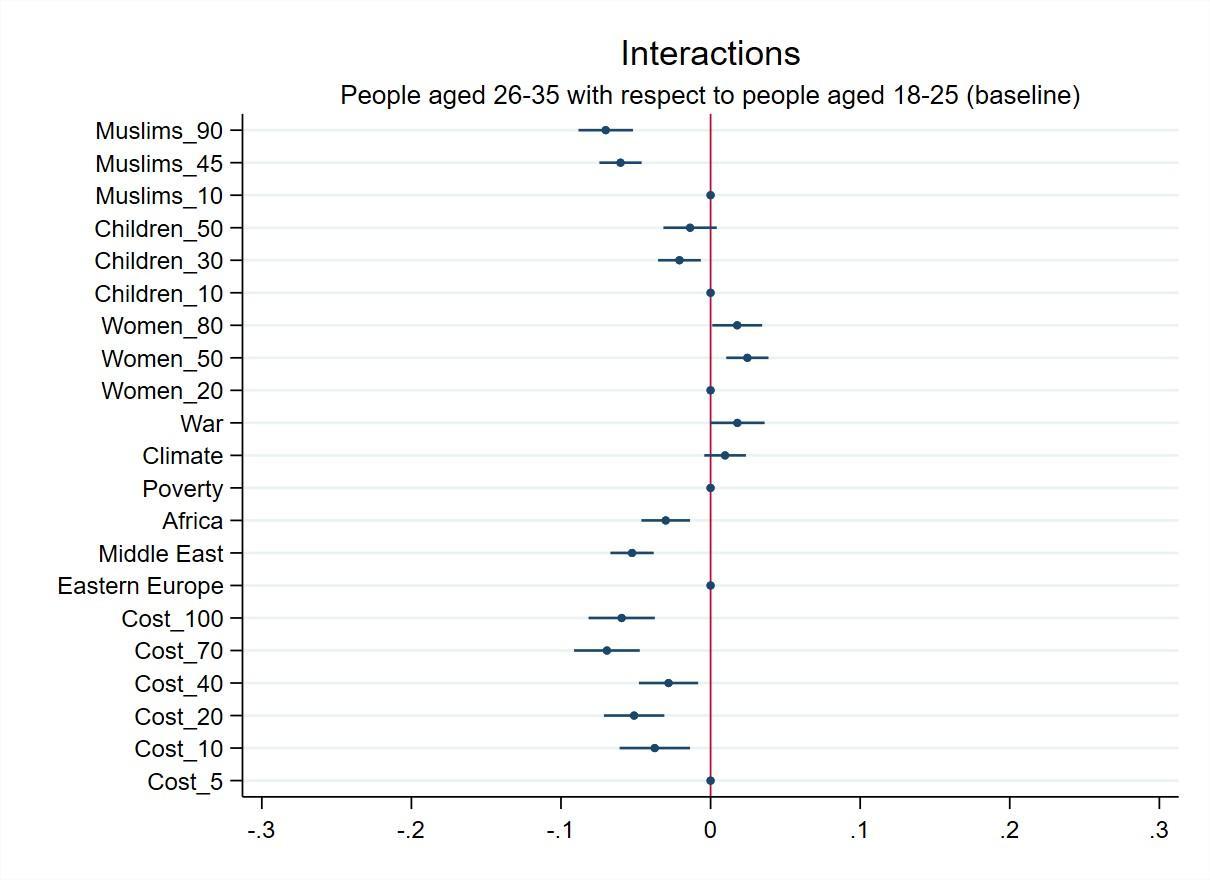


Figure D4a. AMCE with 95% confidence intervals: Age 26-35


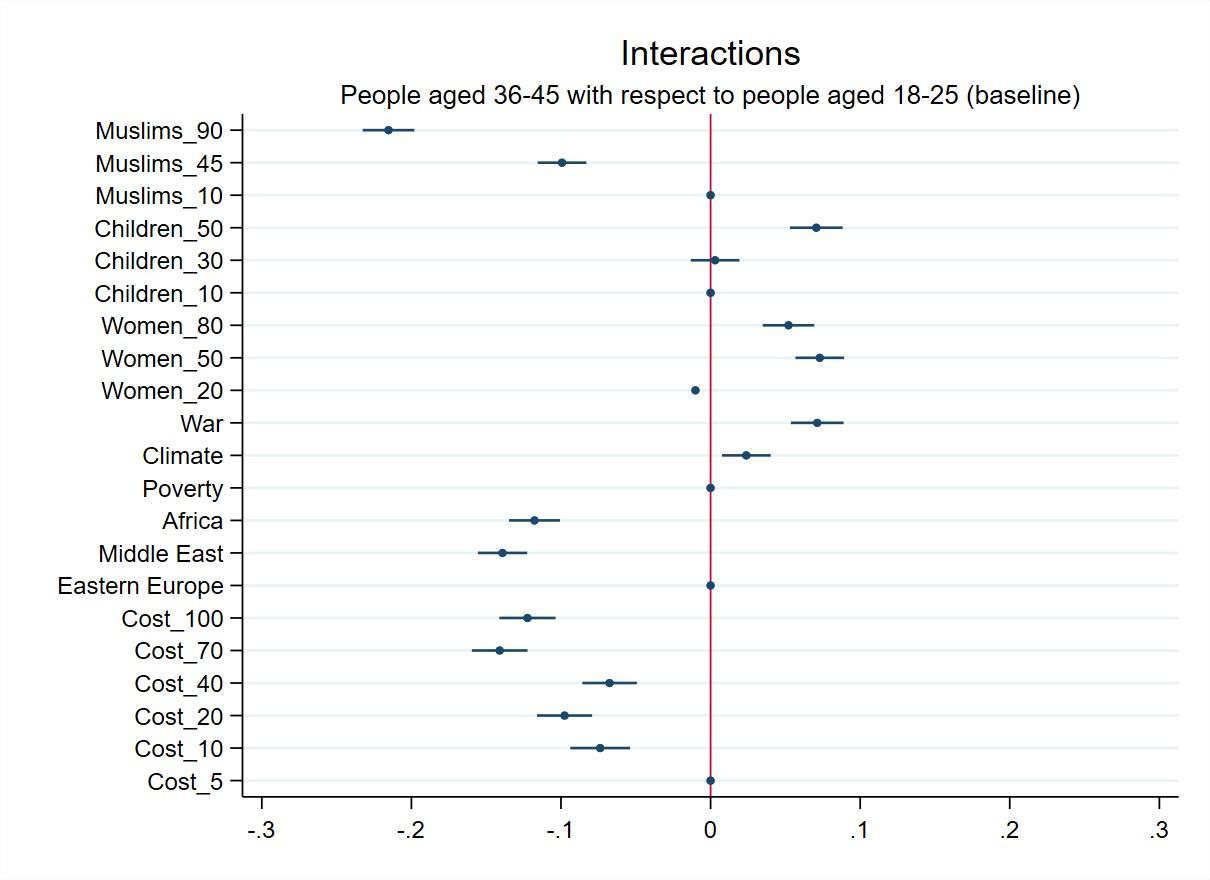


Figure D4b. AMCE with 95% confidence intervals: Age 36-45


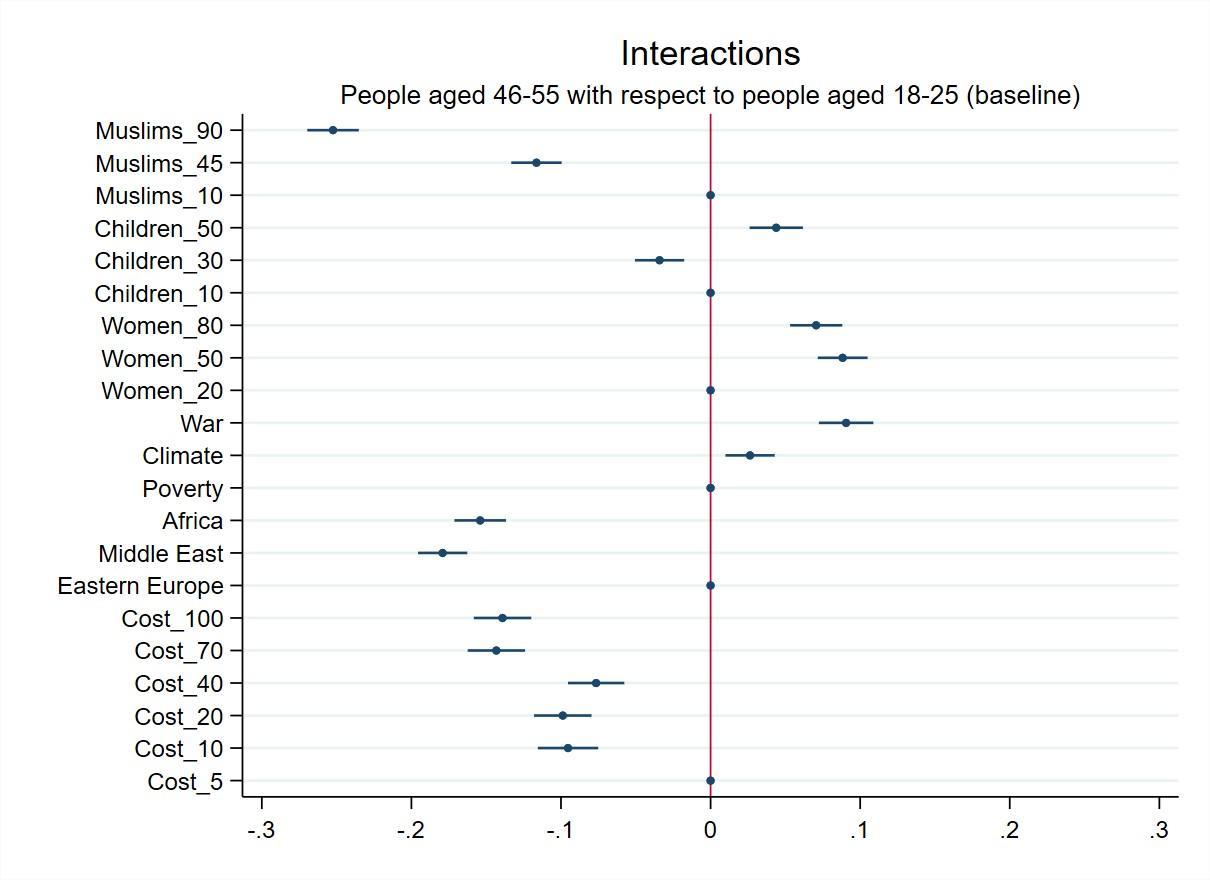


Figure D4c. AMCE with 95% confidence intervals: Age 46-55


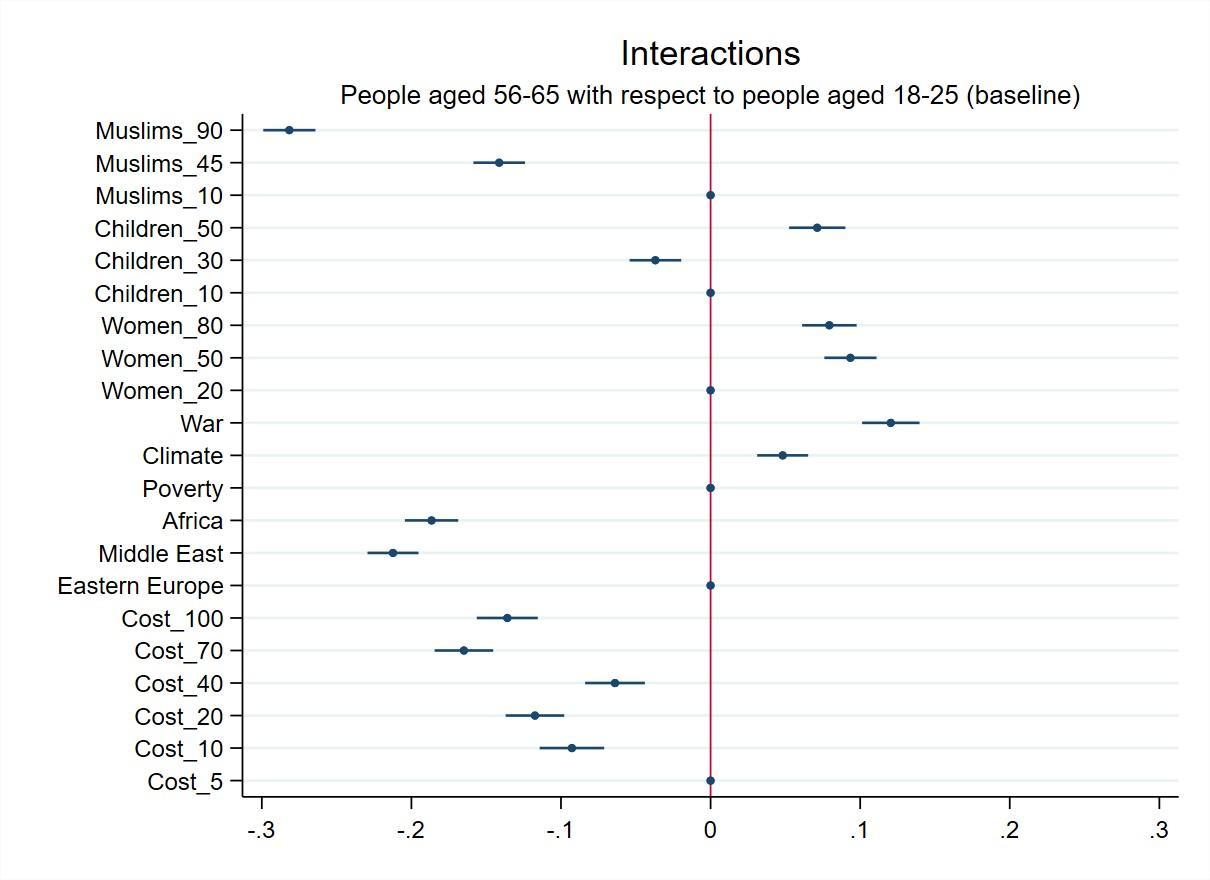


Figure D4d. AMCE with 95% confidence intervals: Age 56-65


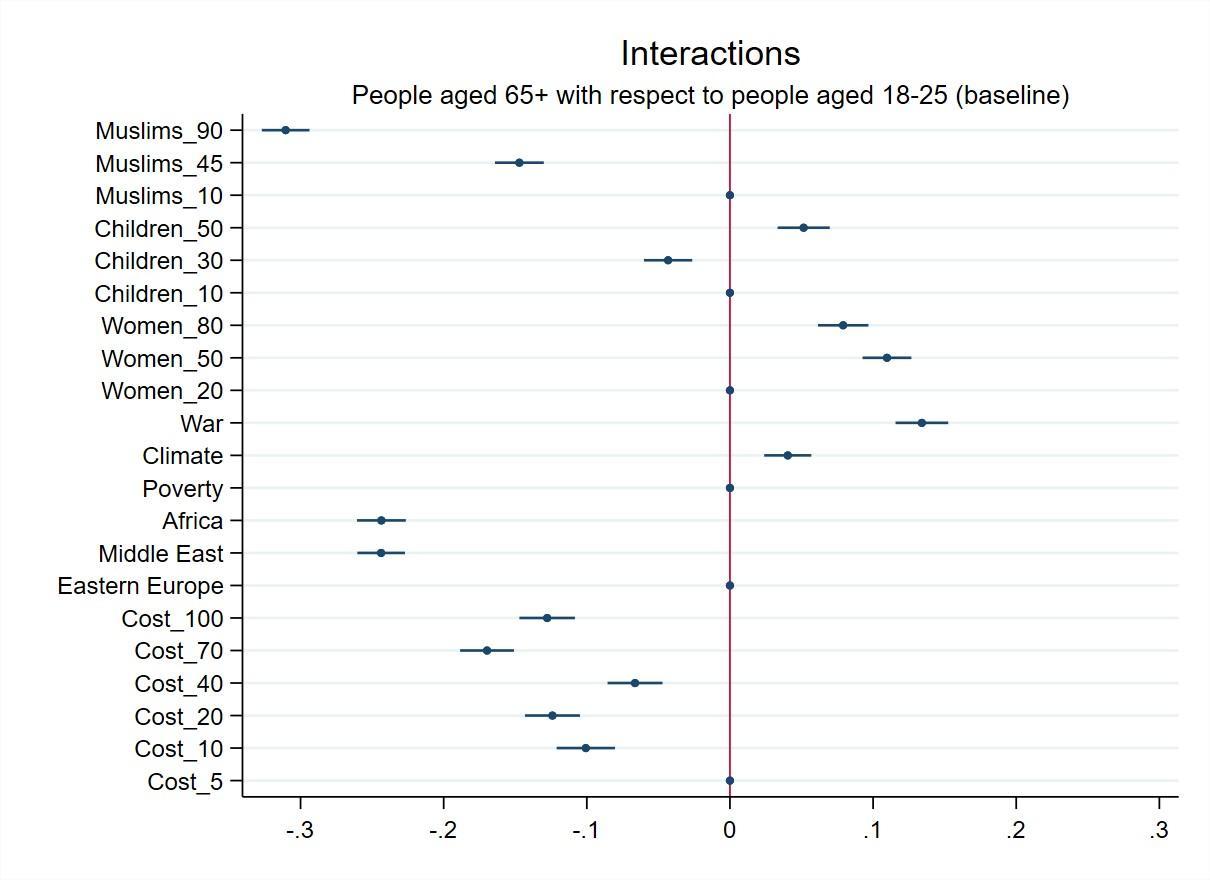


Figure D4e. AMCE with 95% confidence intervals: Age 65+


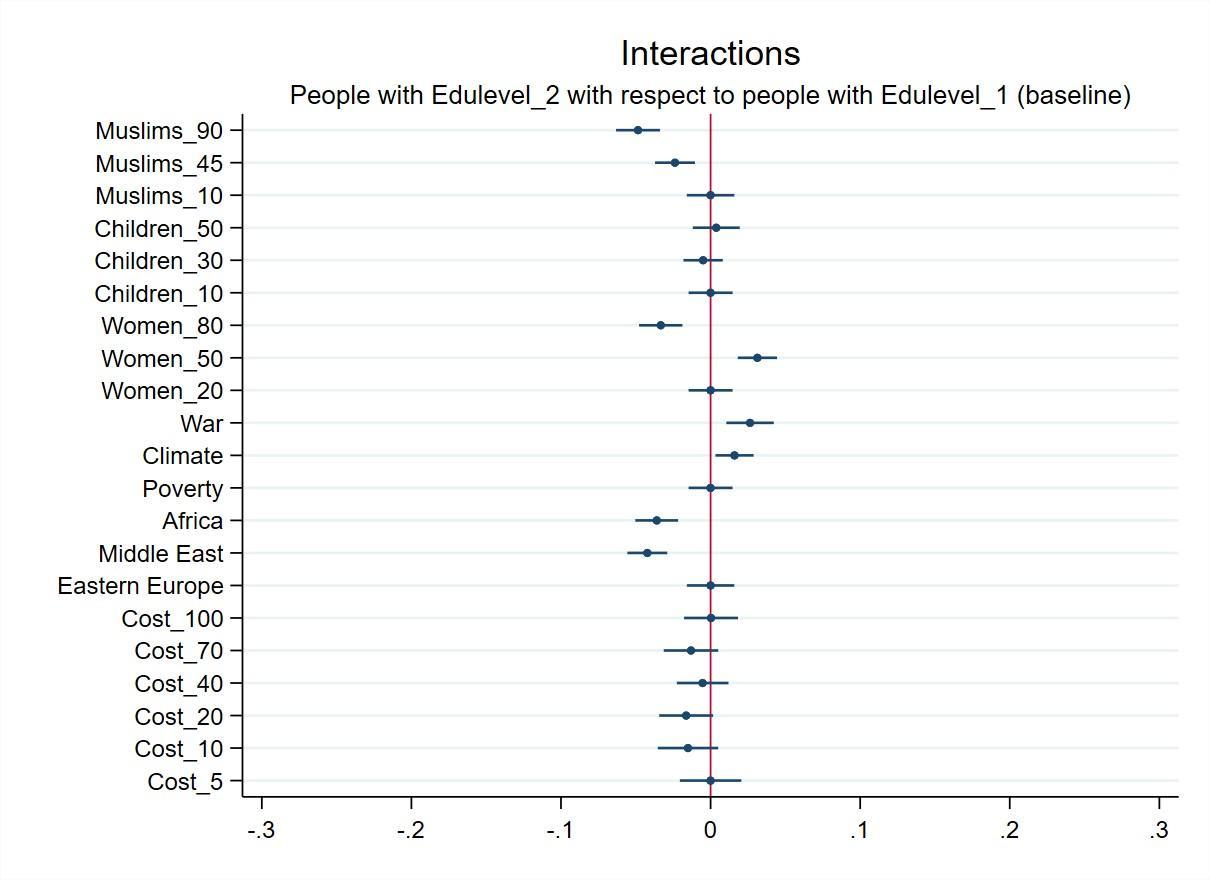


Figure D5a. AMCE with 95% confidence intervals: medium education


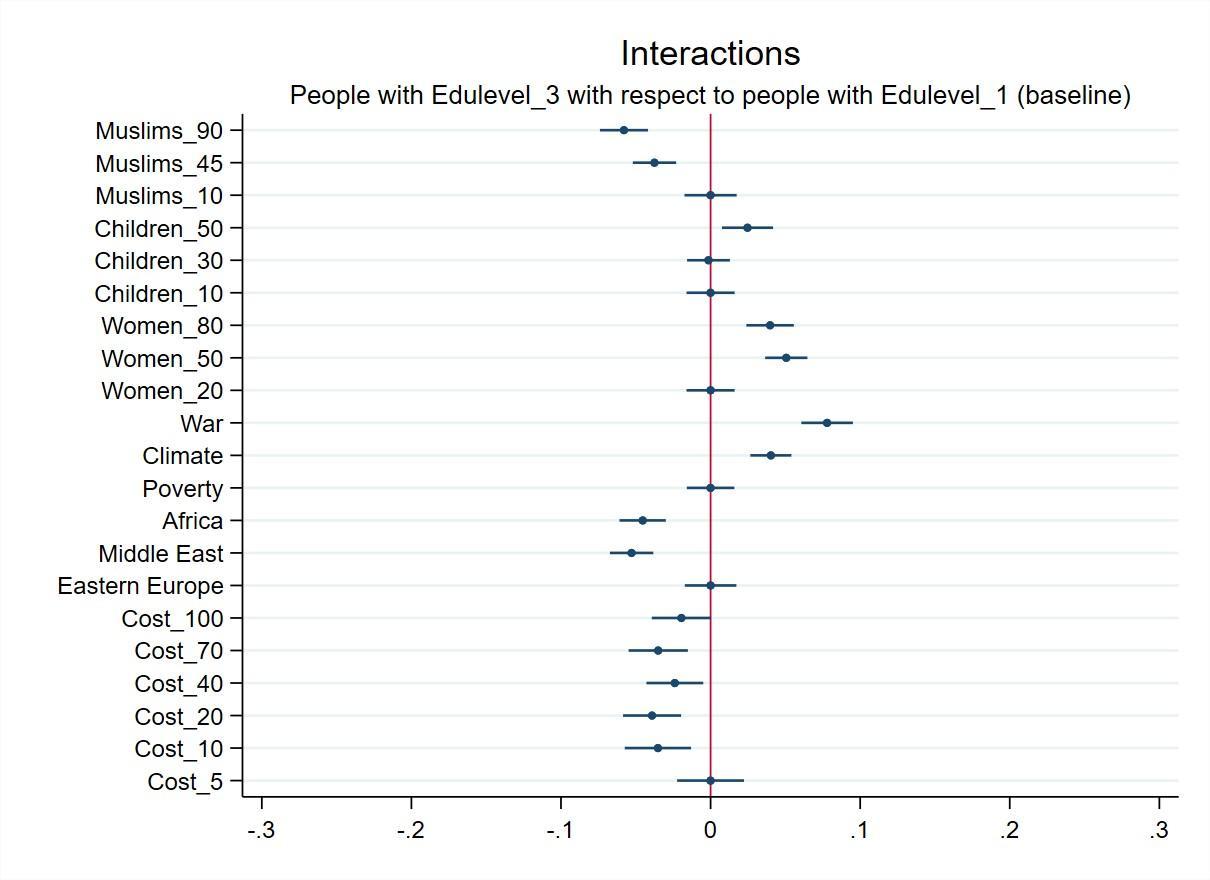


Figure D5b. AMCE with 95% confidence intervals: higher education

Appendix E: Tests of treatment effects

|  |  | |  |  |  |  |  |  |
| --- | --- | --- | --- | --- | --- | --- | --- | --- |
| MEANS | | | | | | | | |
|  | | Treatment1 | | | Treatment2 | | Treatment3 | |
| Variable | | coef. | | st.err. | coef. | st.err. | coef. | st.err. |
| SQ | | -0.496 | | 0.0368 | -0.527 | 0.041 | -0.448 | 0.029 |
| Middle East | | -0.148 | | 0.0128 | -0.143 | 0.013 | -0.127 | 0.010 |
| Africa | | -0.116 | | 0.0082 | -0.122 | 0.009 | -0.105 | 0.007 |
| Climate | | 0.0122 | | 0.0061 | 0.013 | 0.006 | 0.010 | 0.005 |
| War | | 0.106 | | 0.0083 | 0.117 | 0.009 | 0.099 | 0.007 |
| Women_50 | | 0.018 | | 0.0069 | 0.034 | 0.008 | 0.025 | 0.006 |
| Women_90 | | 0.053 | | 0.0056 | 0.067 | 0.006 | 0.053 | 0.005 |
| Children_30 | | 0.043 | | 0.0058 | 0.045 | 0.006 | 0.045 | 0.005 |
| Children_50 | | 0.094 | | 0.0071 | 0.094 | 0.007 | 0.074 | 0.006 |
| Muslim_50 | | -0.077 | | 0.0077 | -0.081 | 0.008 | -0.068 | 0.006 |
| Muslim_90 | | -0.182 | | 0.0154 | -0.176 | 0.016 | -0.156 | 0.012 |
|  | |  | |  |  |  |  |  |
|  | |  | |  |  |  |  |  |
|  | | LL | | K |  | LR_Treatment specific vs. Pooled model | | p-value |
| Pooled model | | -73141.341 | | 24 |  | 57.15 |  | 0.17 |
| Treat1 | | -24403.073 | | 24 |  |  |  |  |
| Treat2 | | -24190.032 | | 24 |  |  |  |  |
| Treat3 | | -24519.663 | | 24 |  |  |  |  |
| Treatment specific joint model | | -73112.767 | | 72 |  |  |  |  |

|  |  | Diff_T1-T2 | | | Diff_T1-T3 | | | Diff_T2-T3 | | |
| --- | --- | --- | --- | --- | --- | --- | --- | --- | --- | --- |
|  | Variable | coef | s.e. | t-stat | coef | s.e. | t-stat | coef | s.e. | t-stat |
|  | SQ | 0.0312 | 0.0550 | 0.57 | -0.0482 | 0.0471 | -1.02 | -0.0794 | 0.0504 | -1.58 |
|  | Middle East | -0.0043 | 0.0184 | -0.23 | -0.0209 | 0.0164 | -1.27 | -0.0166 | 0.0168 | -0.99 |
|  | Africa | 0.0062 | 0.0122 | 0.51 | -0.0108 | 0.0106 | -1.02 | -0.0170 | 0.0113 | -1.50 |
|  | Climate | -0.0008 | 0.0088 | -0.09 | 0.0018 | 0.0081 | 0.23 | 0.0027 | 0.0083 | 0.32 |
|  | War | -0.0109 | 0.0125 | -0.87 | 0.0076 | 0.0108 | 0.70 | 0.0185 | 0.0116 | 1.59 |
|  | Women_50 | -0.0154 | 0.0103 | -1.49 | -0.0067 | 0.0092 | -0.72 | 0.0088 | 0.0099 | 0.89 |
|  | Women_90 | -0.0146 | 0.0085 | -1.71 | -0.0004 | 0.0074 | -0.06 | 0.0141 | 0.0080 | 1.76 |
|  | Children_30 | -0.0021 | 0.0085 | -0.24 | -0.0019 | 0.0078 | -0.25 | 0.0001 | 0.0080 | 0.02 |
|  | Children_50 | 0.0007 | 0.0103 | 0.07 | 0.0202 | 0.0090 | 2.24 | 0.0195 | 0.0093 | 2.09 |
|  | Muslim_50 | 0.0035 | 0.0114 | 0.31 | -0.0094 | 0.0099 | -0.95 | -0.0130 | 0.0104 | -1.24 |
|  | Muslim_90 | -0.0057 | 0.0221 | -0.26 | -0.0251 | 0.0196 | -1.28 | -0.0194 | 0.0200 | -0.97 |

1. More details on this specification can be found in Train, 2009. [↑](#footnote-ref-1)
